# Gene duplication, gene loss, and recombination events with variola virus shaped the complex evolutionary path of historical American horsepox-based smallpox vaccines

**DOI:** 10.1128/mbio.01887-23

**Published:** 2023-09-20

**Authors:** Aline R. V. Souza, Annika Brinkmann, José Esparza, Andreas Nitsche, Clarissa R. Damaso

**Affiliations:** 1 Instituto de Biofísica Carlos Chagas Filho, Universidade Federal do Rio de Janeiro, Rio de Janeiro, Brazil; 2 Centre for Biological Threats and Special Pathogens 1 – Highly Pathogenic Viruses & German Consultant Laboratory for Poxviruses & WHO Collaborating Centre for Emerging Infections and Biological Threats, Robert Koch Institute, Berlin, Germany; 3 Institute of Human Virology, University of Maryland School of Medicine, Baltimore, Maryland, USA; Fondazione Biotecnopolo di Siena, Siena, Italy

**Keywords:** poxvirus, vaccinia virus, variola virus, orthopoxvirus

## Abstract

Vaccinia virus is the active component of all modern smallpox vaccines after the mid-20th century, but it is uncertain to what extent cowpox, vaccinia, and horsepox viruses were used to produce vaccines before then. Genome sequences of six smallpox vaccines used in the United States between 1850 and 1902, namely VK01, VK02, VK05, VK08, VK12, and Mulford_1902 vaccines, revealed >99.5% similarity with a 1976 strain of horsepox in the genome core. However, how these historical vaccines relate to horsepox and vaccinia viruses is still unknown. Here, we present a detailed investigation of the gene content and genomic structure of these historical smallpox vaccines. Except for VK05, all historical vaccines differ from horsepox in the genomic architecture of the flanking variable regions showing complex patterns of gene duplication/transposition, gene fragmentation, and gene loss. The Mulford_1902 vaccine is the closest virus to contemporary vaccinia viruses and the VK02 vaccine is the most different, with several stretches of variola virus genes recombined in its genome. Our data suggest that in the late 19th and early 20th centuries, different horsepox-based vaccines and probably related unsampled progenitors of modern vaccinia virus coexisted. A better understanding of the evolutionary path of the now extinct horsepox-based vaccines will increase our knowledge of the origins of contemporary vaccinia viruses and the pathways that led to the consolidation of current smallpox vaccines. This is particularly important now that the resumption of production of smallpox vaccines for use against mpox is widely discussed, as is the improvement of available vaccines.

**IMPORTANCE:**

Modern smallpox vaccines, such as those used against mpox, are made from vaccinia viruses, but it is still unknown whether cowpox, horsepox, or vaccinia viruses were used in the early 20th century or earlier. The mystery began to be solved when the genomes of six historical smallpox vaccines used in the United States from 1850 to 1902 were determined. Our work analyzed in detail the genomes of these six historical vaccines, revealing a complex genomic structure. Historical vaccines are highly similar to horsepox in the core of their genomes, but some are closer to the structure of vaccinia virus at the ends of the genome. One of the vaccines is a recombinant virus with parts of variola virus recombined into its genome. Our data add valuable information for understanding the evolutionary path of current smallpox vaccines and the genetic makeup of the potentially extinct group of horsepox viruses.

## INTRODUCTION

The smallpox vaccine is one of the most effective vaccines ever developed. It played a pivotal role in the global crusade that led to the declaration of smallpox eradication in 1980. The vaccines used by the World Health Organization smallpox eradication campaign from 1966 to 1980 were based on vaccinia virus (VACV), the prototypic member of the genus *Orthopoxvirus*, family *Poxviridae* (1). Even today, VACV is still the source of smallpox vaccines being used against mpox. However, it is uncertain when VACV started to be used to produce the smallpox vaccine. The history of smallpox vaccines before the mid-20th century remains obscure, as well the origins of VACV itself (2).

History accounts for the use of cowpox virus by Edward Jenner and other vaccinators in the late 18th and early 19th centuries. Yet, Jenner himself and others also reported the use of equine lymph to immunize against smallpox, while other vaccinators empirically mixed pustular material of different origins with the aim to boost the vaccine potency (3 – 8). However, as infection with the orthopoxviruses cowpox, vaccinia, and even horsepox produces similar clinical manifestations in cows, horses, and humans, it is unreliable to determine the origin of smallpox vaccines if it is based solely on the clinical description of the observed pustular disease (3, 5, 9 – 11).

Horsepox virus (HPXV) is the etiologic agent of a pustular disease in horses that is probably extinct nowadays (12, 13). The only existing field strain of HPXV was isolated from wild horses in Mongolia in 1976 (HPXV_MNR-76). The central conserved region of HPXV_MNR-76 and VACV genomes share >98% identity (14), which places HPXV_MNR-76 within the VACV lineage in phylogenetic analyses (15 – 17). On the other hand, HPXV_MNR-76 and VACV genomes differ in the variable regions that flank both sides of the central conserved region. The main difference lies in two insertions of 10.7 kb and 5.5 kb in the HPXV left and right genomic ends, respectively, that are missing in all VACV genomes reported to date (14).

It has been suggested that HPXV is probably the extant virus closest to the ancestor of the VACV lineage (14, 18, 19). However, until recently, the lack of other HPXV genomes in public databases or VACV sequences dated before 1970 has impaired further analyses regarding the evolutionary history of the VACV lineage.

In 2017, our group reported the first genetic evidence for a horsepox-based smallpox vaccine manufactured by Mulford Laboratories in Pennsylvania in 1902, most probably using seed viruses originally imported from Europe. The analysis of the central region of the Mulford_1902 (MFDV_1902) genome revealed >99.7% nucleotide identity with HPXV_MNR-76, whereas the genome ends were structurally similar to those of VACV, i.e., the MFDV_1902 also misses the 10.7 kb and 5.5 kb insertions on the left and right genome ends, respectively (3).

Recently, a report on the partial genomes of five 19th-century smallpox vaccines obtained from vaccination kits from the Mutter Museum in Philadelphia was shown to be horsepox-based vaccines. The dates were estimated to be circa 1859 for VK12, 1866 for VK01, 1873 for VK08, but no date could be estimated for either VK02 or VK05 vaccines (20). Using the archived reads of public database, our group reassembled the full genomes of the five historical vaccines and showed that VK05 probably represents a bona fide HPXV, whereas the structures of the genomes’ ends of the other four vaccines (VK01, VK02, VK08, and VK12) differ (16). However, a comprehensive analysis of the relationship between the genomes of VK viruses and the MFDV_1902 has not been done. How these historical vaccines relate to HPXV_MNR-76 and VACV is still unknown.

Therefore, here, we analyze the variable ends and the central conserved regions of the genomes of six historical smallpox vaccines used in the United States between 1850 and 1902. We investigate their gene set and genome structure that reveal a complex pattern of gene duplication/transposition, gene fragmentation, and gene loss, as well as the occurrence of genetic recombination events with variola virus (VARV). Our analysis sheds light on the origins of contemporary VACV and suggests that they may have evolved from viruses closely related to the VK and MFDV_1902 vaccines. These putative progenitors of modern VACV probably coexisted with ancient HPXV-based vaccines. Our study may guide future studies on the evolution of smallpox vaccines and the origins of modern VACVs.

## MATERIALS AND METHODS

### Genome sequences

Genome sequences from the historical smallpox vaccines analyzed in this study have been previously determined and annotated. They are publicly available in the GenBank Database under the following accession numbers: Mulford_1902: MF477237 (3) and the reassembled genomes of VK01: BK013339, VK02: BK013340, VK05: BK013341, VK08: BK013342, and VK12: BK013343 (16, 20). Other genome sequences included in this study are listed in Table S1.

### Multi-sequence alignments

We first generated good quality alignments for the three main genome regions analyzed in this study: the left and right variable ends of the genomes and the central conserved regions. The central region (it encompasses the orthologs of VACV_Cop F9L gene to A24R gene) was aligned using the Mafft server v.7 with default parameters (21). Because of the complex genomic structure of the variable ends (it encompasses all genes to the left of F9L and all genes to the right of A24R), these regions were extracted from the genomes and aligned using Geneious alignment (Geneious Prime v 2021.0.3), opting for the multiple global alignment tool with free end gaps and stringent parameters (cost matrix 93% similarity, gap open penalty 100, gap extension penalty 0.05 with two refinement iterations). For whole-genome alignments, we concatenated the alignments obtained for the conserved and variable regions using Geneious Prime v 2021.0.3. All alignments were visually inspected using either CLC Main Workbench v20.0.4.1 or Geneious Prime v 2021.0.3 and manually edited if necessary. Pairwise comparison was created using CLC Main Workbench v20.0.4.1 and values were transferred to Microsoft Excel to generate a similarity heat map. To investigate polymorphic sites, the alignments were analyzed using Base-By-Base v3 (22) to scan for single nucleotide polymorphisms (SNPs), insertions, and deletions (INDELS).

The genomic architecture of the variable ends was investigated using CLC Main Workbench v20.0.4.1. Alignments were zoomed out to omit minor deletions (<250 bp) and highlight major patterns of interest. The resulting linear forms of the alignments were used as scaffolds for drawing schemes of the genomic structure using CorelDraw standard 2021 v.23.0.0.363. For a detailed analysis of specific regions, the alignments were zoomed in, and the regions of interest were analyzed in the annotated genome files using CLC Main Workbench v20.0.4.1.

### Analysis of gene set and patterns of gene fragmentation

We built a table comparing the annotated genes of several genomes of interest and used it to select the genes in the telomeric regions. These regions included the left end of the genomes upstream VACV-Cop C9L ortholog, and the right end of the genome downstream VACV_Cop B9R ortholog. Genes were computed if they had ≥30% of the size of its ortholog in HPXV_MNR-76; duplicated genes in the inverted terminal repeats (ITRs) were counted only once. This table was used as input to construct a Venn diagram (http://bioinformatics.psb.ugent.be/webtools/Venn/) that was modified manually to insert dual diagrams using CorelDraw standard 2021 v.23.0.0.363. The comparative annotation table was also used to identify the patterns of gene fragmentation along the genomes.

### Recombination analysis

Sequence alignment data sets were used to generate similarity plots using Simplot v.3.5.1 (23), opting for Kimura-2p and Gapstrip on. Values of window size and step size are specified in figure legends. Putative recombination regions were analyzed using the Bootscan tool implemented in Simplot v.3.5.1 with the same parameters described above and the neighbor-joining tree model. The regions of the alignments between recombination breakpoints were extracted using Geneious Prime v 2021.0.3 and used to reconstruct the phylogenetic tree as described below.

### Phylogenetic analysis

The whole-genome alignment of 48 orthopoxvirus sequences, which represents the main species of orthopoxviruses and the vaccinia lineage, was trimmed to select approximately 20 kb of each variable region flanking both sides of the central conserved region, resulting in an alignment of nearly 150 kb long. Phylogenetic analysis was conducted using MEGA X (24) opting for the maximum likelihood method, GTR model, NJ/BioNJ input tree, five-category discrete G + I, complete deletion, and 1,000 bootstrap replicates. Phylogenetic analyses of putative recombinant regions in VK02 genome were based on aligned sequences extracted from the regions within recombination breakpoints. Trees were constructed as described above and confirmed by using the neighbor-joining method with Kimura-2p or Tamura-Nei models, opting for five-category discrete gamma distribution, complete deletion, and 1,000 bootstrap replicates.

## RESULTS

### Genetic diversity of the historical American smallpox vaccines

**Fig 1:**
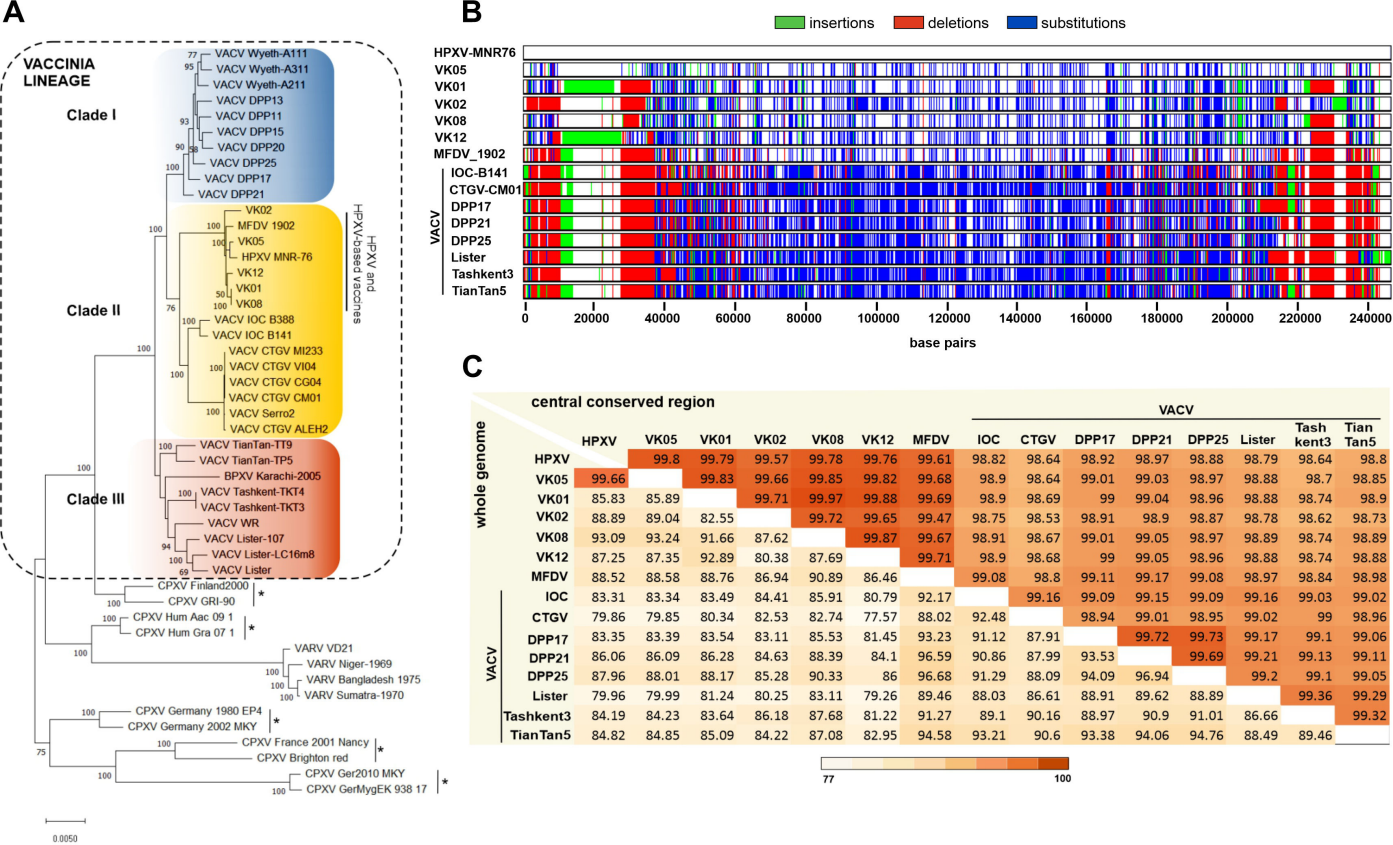
Phylogenetic tree and genome relationships of the historical American smallpox vaccines and HPXV_MNR-76. (**A**) Multialignment of 48 orthopoxvirus sequences was used as input for a tree built using the maximum likelihood model implemented in MEGA X, as described in Materials and Methods. The three clades of the VACV lineage (dotted box) are colored. The subclade containing HPXV and the old smallpox vaccines is indicated on the right of the vaccinia lineage. Asterisks indicate the five clades of cowpox virus (CPXV) strains. Numbers indicate the percentage of bootstrap support from 1,000 replicates (>50% is shown). The scale bar indicates the number of substitutions per site. (**B**) Base-by-base profile of nucleotide substitutions (blue), deletions (red), and insertions (green) along the whole genomes of HPXV_MNR-76 (used as reference genome), the historical vaccines, and different VACV strains. (**C**) Identity heat map generated by pairwise comparison of the whole genome (left half panel) and the central conserved region (right half panel) of the historical smallpox vaccines, HPXV_MNR-76, and eight VACV strains.

Previous reports have suggested that the historical smallpox vaccines from the Mutter Museum, namely, VK01, VK02, VK05, VK08, and VK12, and from the Mulford Laboratory, MFDV_1902, were closely related to the Mongolian horsepox virus strain MNR-76 (3, 16, 20). In fact, it was suggested that the VK05 vaccine represented a bona fide HPXV strain (16). The phylogenetic tree, based on a 150 kb extended central region of 48 orthopoxvirus genomes, confirms previous data showing the three-clade topology of the VACV lineage (Fig. 1A). All historical vaccines and the HPXV_MNR-76 cluster in a subclade of clade II that also includes the Brazilian VACVs (16, 20). However, HPXV also shares a close relationship with its sister clade I, which includes the VACV Wyeth strain and clones of the American Dryvax vaccine (15, 17).

It is important to note that phylogenetic diagrams depict the genetic relatedness between viruses but other important genomic features to understand the evolutionary path of the VACV lineage are eclipsed in this type of analysis, such as INDELs, duplications/translocations, and recombination events in microregions of virus genomes. Therefore, to investigate in detail the relationships among members of the HPXV subclade, the full genome sequences of the ancient vaccines, together with HPXV_MNR-76 and different modern VACV strains, were aligned and screened for SNPs and INDELs. Figure 1B shows that all historical vaccines, including the bona fide HPXV strain VK05, accumulated variations in their genomes in relation to the reference genome HPXV_MNR-76. However, they clearly form a related group apart from the modern VACV strains from the three clades, which overall accumulated more variations than did the historical vaccines in relation to HPXV_MNR-76, as expected.

**Fig 2:**
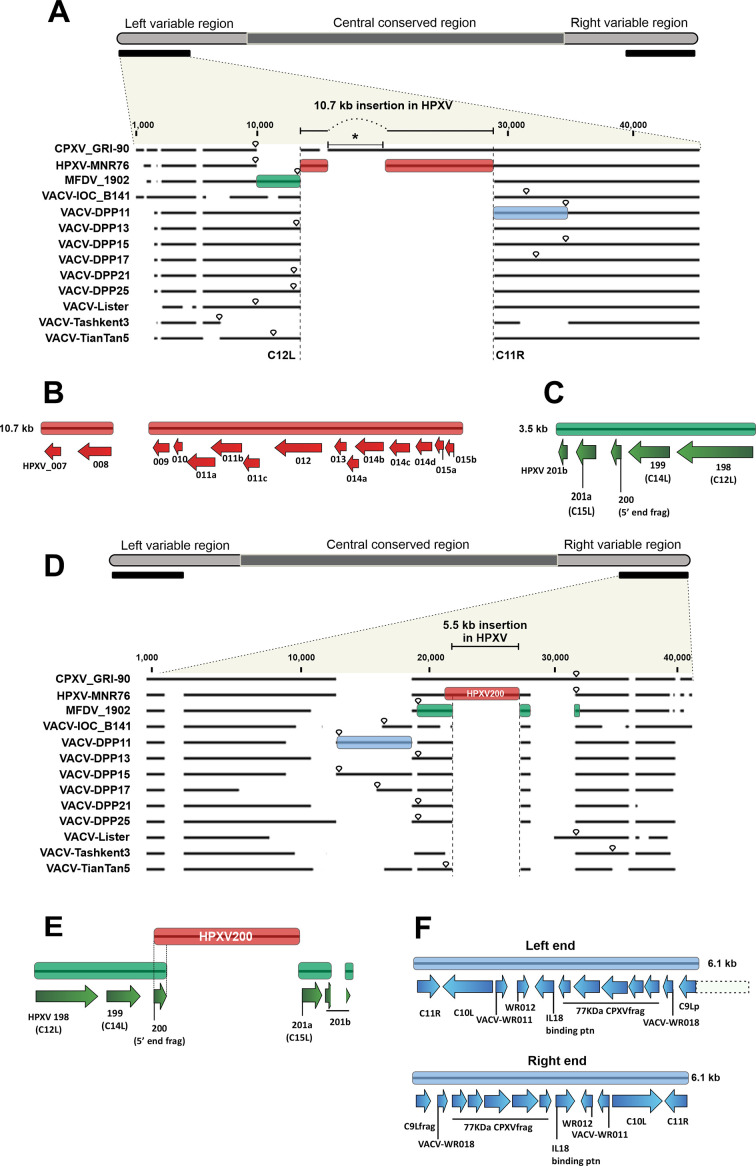
Comparing the genomic architecture of the left and right variable regions of the MFDV_1902 smallpox vaccine with CPXV, HPXV_MNR-76, and VACV strains. Multialignments of the initial and final 40 kb segment of the virus genomes were used as scaffold for drawing schematic diagrams. Top panels in panels A and D show the general scheme of orthopoxvirus genomes, indicating the regions analyzed in the alignments shown in the bottom panels. Bottom panels in panels A and D: the 10.7 kb and 5.5 kb insertions, respectively, at the left and right ends of the HPXV_MNR-76 genome are indicated at the top of the alignments. Inverted teardrops indicate the ITR boundaries. (B) The HPXV_MNR-76 genes present in the red box in panel A. (C and E) The genes present in the green boxes represented in panels A and D, respectively. Genes are identified as HPXV_MNR-76 orthologs or VACV_Cop orthologs (in parentheses). (F) The genes identified as their VACV_Cop or VACV_WR orthologs present in the blue boxes in panels A and D. Note that all aligned regions have the same genes. For the sake of clarity, boxes have not been drawn in all aligned sequences. The dotted lines represent the part of the genes that are not included in the regions of interest.

The similarity heat map, shown in Fig. 1C, confirms the results of phylogenetic inference (Fig. 1A) and supports the accumulation of polymorphic sites in the genomes shown in Fig. 1B. The central conserved regions of the genomes of the VK viruses, MFDV_1902, and HPXV_MNR-76 are strikingly similar, as expected, as they encode essential proteins for poxvirus replication. The lowest level of similarity was 99.47% between the MFDV_1902 vaccine and the most distant VK02, and the highest similarity score (99.8%) was shared between VK05 and HPXV_MNR-76 (Fig. 1C). However, analysis based on whole-genome alignment, which includes the central regions plus the flanking variable regions, as schematized in the top panel of Fig. 2A, led to substantially lower levels of identity among the historical vaccines, reaching a low level of 80.38% (Fig. 1C). Yet, VK05 remained highly similar to HPXV_MNR-76 (99.66%), and MFDV_1902 maintained high identity with some VACV strains (>96%) even when the whole genome was analyzed. The left and right variable regions of poxvirus genomes harbor genes involved in host range, immunomodulatory functions, virulence, and virus-host interactions; here, they will be collectively referred to as accessory genes, as suggested by Senkevich et al. (25).

### The variable ends of the Mulford_1902 genome share similar patterns of gene duplication/ transposition and gene loss with VACV strains

To investigate the genomic architecture and gene content of the historical vaccines, we first analyzed the variable ends of the genomes. Preliminary analysis of the genome ends of the MFDV_1902 suggested that they are less complex than those of the VK viruses and more structurally close to VACV genomes (3). Therefore, for the sake of clarity, we examined the MFDV_1902 variable terminal regions separately from those of the VK viruses, to identify the main consensual genomic features that have been used in several studies so far to distinguish HPXV from all contemporary VACV strains (14, 15, 17, 26). This and all subsequent analyses included representative strains of VACV from the three clades that were used as a smallpox vaccine. We also analyzed a CPXV genome of the VACV-like clade (CPXV strain GRI-90) because of the similar genome architecture of HPXV and CPXV genomes, as previously reported (14).

The initial 40 kb segment at the left end of the alignment is shown in Fig. 2A. It confirms that, similar to all VACV strains, MFDV_1902 also lacks the main genomic feature that separates HPXV from VACV, i.e., the 10.7 kb stretch of genes present in the HPXV_MNR-76 genome (Fig. 2A, red boxes). This insertion aligns between the VACV orthologs of the C12L and C11R genes (based on the nomenclature of VACV-Cop) in a region close to the boundaries of the ITRs (Fig. 2A, inverted teardrop). The 10.7 kb insertion harbors 15 open reading frames (ORFs) annotated as 15 individual genes in the HPXV_MNR-76 genome (Fig. 2B). As previously reported, CPXV also preserved this region (Fig. 2A), which is not surprising. Among all extant orthopoxviruses, CPXV is believed to be most similar to what was once the ancestor of the genus *Orthopoxvirus* due to its nearly complete gene repertoire (26 – 30). However, compared to HPXV, CPXV has nine intact accessory genes in this segment; most of them encode ankyrin proteins (Fig. S1A, genes highlighted in red). Thus, some genes annotated in the HPXV insertion, in fact, represent fragments of intact CPXV genes. For example, the CPXV_GRI-90 D14L gene corresponds to HPXV genes 011a to 011c (Fig. 2B; Fig. S1A, genes highlighted in red). Furthermore, CPXV has a 4.7 kb sequence that is missing from the HPXV genome (Fig. 2A, asterisk).

The regions flanking the 10.7 kb deletion in the VACV and MFDV_1902 genomes seem to be hotspots for genomic alterations. A 3.5 kb sequence upstream this deletion (green box in Fig. 2A) is found in MFDV_1902 and in several modern VACV strains but is missing in VACV_Tashkent and in both HPXV_MNR-76 and CPXV genomes. The region has five accessory genes (Fig. 2C), including the serine protease inhibitor-like SPI-1 (VACV_Cop C12L ortholog). These genes form a syntenic sequence on the right ends of the HPXV_MNR-76 and CPXV genomes but are missing on their left ends. Likewise, they are found on the right ends of VACV and MFDV_1902 genomes, as discussed below (green boxes in Fig. 2D). CPXV, which is believed to be more similar to an ancestor of the *Orthopoxvirus* genus, has these genes only at the right end of its genome; therefore, it seems that the right ends of the VACV and MFDV_1902 genomes originated the duplication/transposition of the five accessory genes seen at the left end.

Figure 2D shows the alignment of the final 40 kb segment on the far-right end of these genomes. We observe that MFDV_1902, like all VACV, has nearly lost the entire HPXV200 gene (the 5.5 kb insertion in HPXV genome), which is another genomic hallmark that distinguishes contemporary VACV strains from HPXV (Fig. 2D, red box). Indeed, MFDV_1902 and most VACV strains still retain a remnant 200 bp fragment of the 5´ end of the HPXV200 gene (Fig. 2E). Like HPXV_MNR-76, all CPXV genomes have an intact HPXV200 ortholog (Fig. 2D and Fig. S1A).

The remnant fragment of the 5´ end of the HPXV200 gene retained by the MFDV_1902 and VACV genomes is also found at the left end of the genome as part of the duplicated 5-gene sequence (Fig. 2A, green box; Fig. 2C). Interestingly, the remnant fragment was duplicated/transposed contiguously to the VACV C15L gene (HPXV201a ortholog), which in the HPXV_MNR-76 genome is the next gene downstream of HPXV200 (Fig. 2C and E). This finding suggests that both MFDV_1902 and modern VACV strains lost the HPXV200 gene before the right-to-left duplication/transposition event took place in the evolutionary path of the VACV lineage.

The regions that flank the 5.5 kb deletion on the right end of the genome of the MFDV_1902 and VACV strains seem to be hotspots for genomic instability and alterations. Upstream of the deletion in some VACV strains, but not MFDV_1902, is an insertion of 12 or fewer genes (Fig. 2D, blue boxes; Fig. 2F) that were probably duplicated/transposed from the left end to the right end (Fig. 2A, blue box). VACV genomes bearing that duplication are diploid for important accessory genes, such as the VACV C11R ortholog, which encodes the secreted VACV epidermal growth factor, and VACV C10L, which encode both an interleukin-1 (IL-1) receptor antagonist and a TANK-binding kinase 1 (TBK1) inhibitor (25, 31). The fact that this duplication event is only found in a few VACV strains suggests that it is a more recent event in the evolutionary path of some VACV strains.

### The variable genome ends of VK viruses share some structural genomic features with HPXV and have long gene insertions

**Fig 3:**
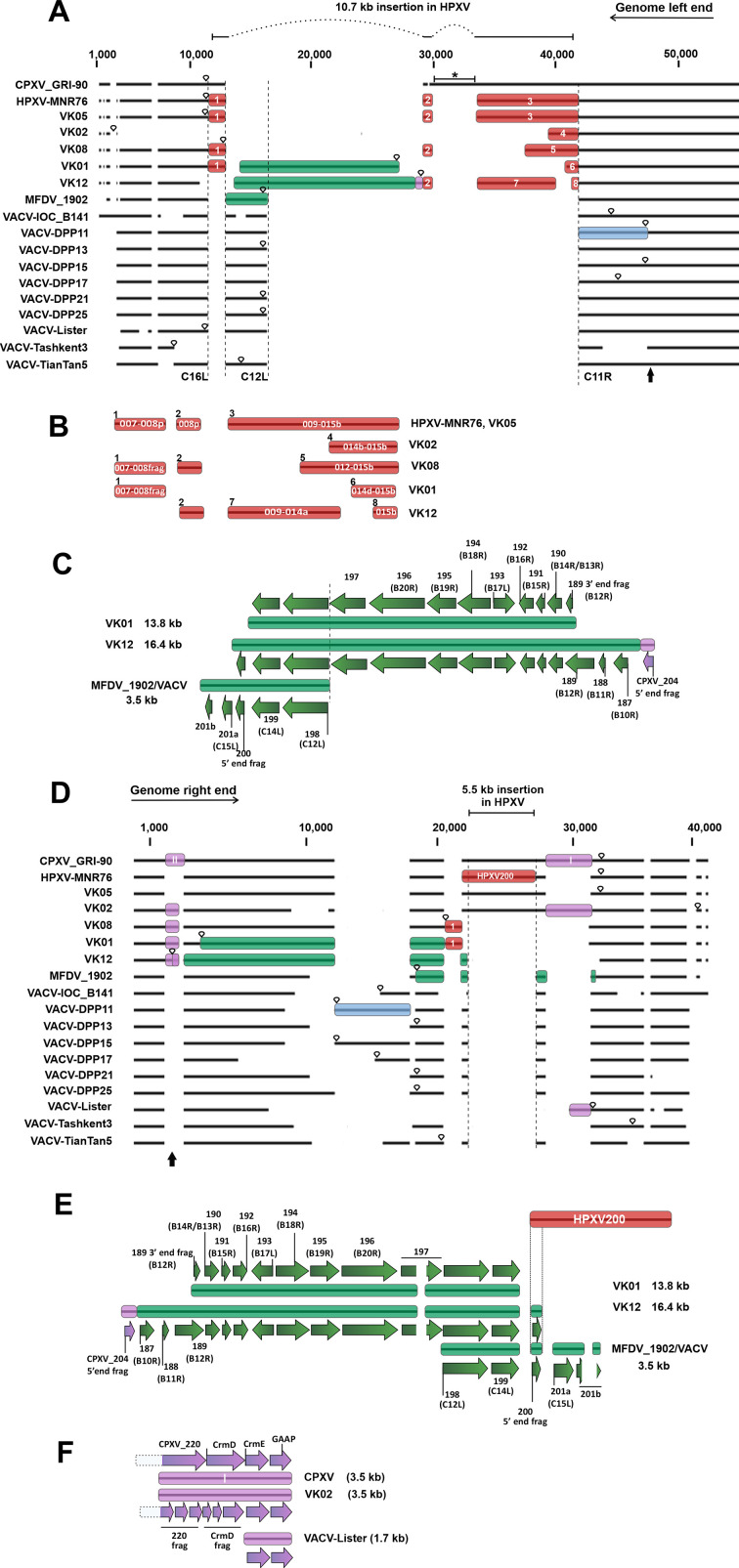
Comparing the genomic architecture of the left and right variable regions of the historical American smallpox vaccines with CPXV, HPXV_MNR-76, and VACV strains. Multialignments of the initial 50 kb and final 40 kb segments of the virus genomes were used as scaffold for drawing schematic diagrams of left and right ends, respectively, in panels A and D. The regions analyzed in these alignments are indicated as black bars in the top panels of Fig. 2A and D. The 10.7 kb and 5.5 kb insertions, respectively, at the left and right ends of the HPXV_MNR-76 genome are indicated at the top of the alignments. Inverted teardrops indicate the ITR boundaries. The black arrows indicate the ortholog of C9L in panel A and the ortholog of B9R in panel D, which were defined as the boundaries of the hypervariable regions used for the gene content analysis. Genes located in the blue boxes are shown in Fig. 2G. (B)HPXV_MNR-76 genes present in the red boxes numbered from 1 to 8 in panel A. (C and E) Genes identified as HPXV_MNR-76 orthologs or VACV_Cop orthologs (in parentheses) present in the green boxes represented in panels A and D, respectively. (F)CPXV orthologs present in purple boxes I in panel D. Note that all aligned regions have the same genes. For the sake of clarity, boxes have not been drawn in all aligned sequences. The dotted lines represent the part of the genes that are not included in the regions of interest.

Adding VK genomes to the alignments increased the complexity of the variable-end architecture. The same genome regions analyzed in Fig. 2 were explored in Fig. 3. Figure 3A shows the initial 50 kb segment of the alignment of the left ends of the genomes. The red boxes represent the 10.7 kb insertion in HPXV_MNR-76. Like HPXV_MNR-76, the VK05 virus retains the full 10.7 kb insertion at the left end, but the other four VK viruses lost pieces of different sizes of this insertion and at different locations, as shown in Fig. 3B. It is noteworthy that VK02 lost all genes upstream of the HPXV_014b ortholog, resulting in the shortest ITR of all ancient smallpox vaccines with only 616 bp (16).

Interestingly, the left ends of VK01 and VK12 genomes, similarly to those of the MFDV_1902 and VACV strains, harbor a gene stretch that is not found at the left end of the other three VK genomes, HPXV_MNR-76, or CPXV genomes (Fig. 3A, green boxes; details in Fig. 3C). These gene stretches are much longer than those of the MFDV_1902 and VACV strains, which have 3.5 kb. In VK01 and VK12 genomes, the stretches have 13.8 kb and 16.4 kb, respectively (Fig. 3C). To rule out genome misassemble, the read mapping was rechecked to confirm adequate read density on the long insertions and near their boundaries, as well as the accuracy of the read mapping at the boundaries (Fig. S2). In VK01, this long segment harbors 11 accessory genes, while the stretch in VK12 has 15 accessory genes (Fig. 3C). The genes in these segments are also located in syntenic position on the right end of the genomes of all ancient vaccines (partially in VK02), HPXV_MNR-76, CPXV, and several VACV strains (Fig. 3D, green boxes; details in Fig. 3E). Therefore, it is reasonable to assume that the long gene stretches on the left end of VK01 and VK12 genomes were probably duplicated/transposed by nonhomologous recombination-driven events from the right to the left end. As a consequence, important accessory genes, such as the orthologs of VACV B19R (the soluble interferon alpha/beta (IFN α/β) receptor), B16R (the soluble IL-1β receptor), B13R/B14R (SPI-2/CrmA apoptosis inhibitor), C12L (serine protease inhibitor-like SPI-1) (25, 31), and others (Fig. 3C and E) are diploid in the VK01 and VK12 genomes.

These long stretches in VK01 and VK12 change the topology of the alignment of the left terminal region that we initially saw in Fig. 2A. In VK01, we observe a shift of HPXV gene 007 and a fragment of HPXV008 to the left of the 13.8 kb insertion (Fig. 3A and B, red box 1), which may have occurred concomitantly with the right-to-left duplication/transposition of this insertion. However, in VK12, MFDV_1902, and modern VACV strains, transposition of the duplicated regions possibly led to the complete loss of HPXV genes 007 and 008, in a concomitant, or subsequent, event. In the case of VK12, similarly to what is seen in VK08, a 29-nt fragment of the HPXV008 gene still remains in the genome (Fig. 3B, box 2).

Analysis of the variable regions at the right ends of these genomes reveals that all VK viruses, except VK05 and VK02, lost the HPXV200 gene (Fig. 3D). Thus, VK05 represents a *bona fide* HPXV strain with all genomic characteristics found on the HPXV variable ends. In fact, HPXV_MNR-76 and VK05 preserve the overall genome architecture of all CPXV strains but share higher nucleotide identity rates with contemporary VACV strains than with CPXV strains of the five clades (Fig. S1A and B).

As for VK02 virus, its genome not only retained an intact copy of HPXV200 but also some CPXV genes, namely CPXV_220, and the genes encoding the tumor necrosis factor receptor II homologs (CrmD and CrmE) and the Golgi antiapoptotic protein (GAAP) (25, 31), although in the VK02 genome, CPXV_220 and CrmD are disrupted (Fig. 3D, purple box I, and Fig. 3F). In contrast to other modern VACV strains but similar to VK02, Lister-derived VACV strains also retain CPXV CrmE and GAAP (Fig. 3D, purple box I and Fig. 3F). Other CPXV DNA segments that contain CPXV_203 gene were retained by VK01, VK02, VK08, and VK12, but not VK05, HPXV_MNR76, MFDV_1902, or any modern VACV (Fig. 3D, purple box II).

Other genomic duplications and transpositions are observed in the right terminal region of the VK01 and VK08 genomes. An intact HPXV007 and a fragment of HPXV008 orthologs are located upstream the 5.5 kb deletion (Fig. 3D, red box 1). These events support the idea that this region is an important hotspot for genomic alterations in the HPXV subclade and, in general, in the VACV lineage.

### Telomeric gene set and gene fragmentation patterns in the genomes of historical smallpox vaccines

The analysis of the gene set found in the genomes’ ends of the historical vaccines reveals that just a few novel genes have been detected in these regions when compared with HPXV_MNR-76. For this analysis, we considered all genes located on the left end upstream of the ortholog of VACV C9L and all genes on the right end downstream of the ortholog of VACV B9R (Fig. 3A and D, indicated by the black arrow on the bottom). The telomeres of these genomes are regions of hypervariability in the historical vaccines due to the numerous gene insertions, deletions, and transpositions. For each genome, we computed only genes ≥30% of the size of their orthologs in HPXV_MNR-76; duplicate genes were counted only once. Considering the six vaccines and HPXV_MNR-76, we computed a total of 48 genes, of which 43 were found in both HPXV_MNR-76 and VK05. Therefore, both viruses have the same gene set in their telomeric regions. VK02 has 38 genes in the analyzed region, VK12 has 36, VK08 has 35, VK01 has 30, and MFDV_1902 has 27 genes. Each of the 43 genes in these hypervariable regions of HPXV/VK05 is represented at least in one of the five vaccine genomes, and all of them are present in CPXV genomes, which have a total of 52 genes in these telomeric regions (Fig. S1C).

**Fig 4:**
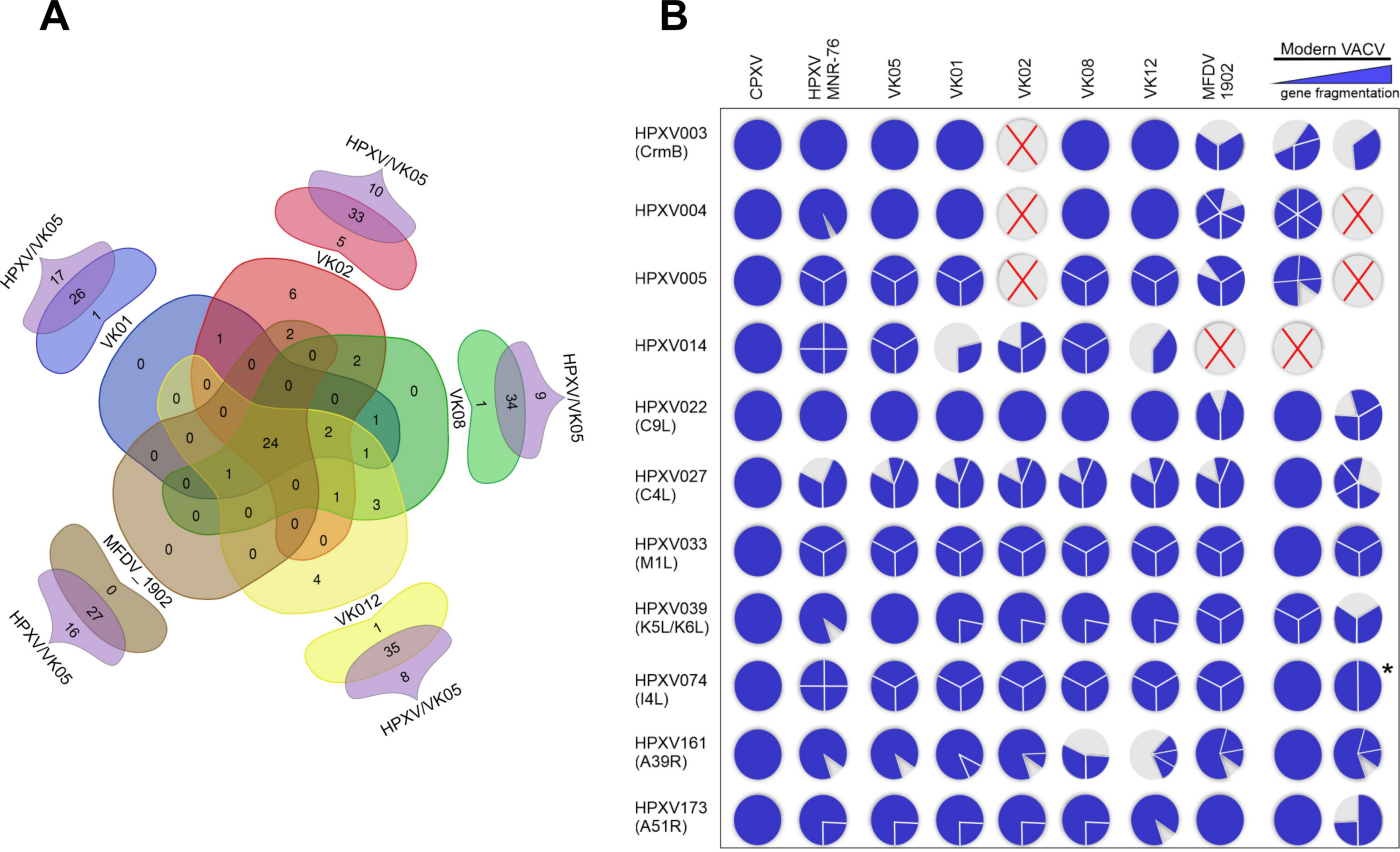
Gene content and patterns of gene fragmentation in the genomes of historical smallpox vaccines. (**A**) Mandala-shaped Venn diagram. The five-set center of the diagram shows the gene counts found in the hypervariable regions of the genomes shared between VK01, VK02, VK08, VK12, and MFDV_1902 vaccines. The dual-set floating diagrams surrounding the central mandala show gene counts shared by HPXV_MNR-76/VK05 and each of the five historical vaccines. (**B**) Schematic representation of the fragmentation patterns of selected genes shown in the Table S1. Each gene is represented by a circle with a gray background and blue parts when the gene exists in virus genomes. The gray circles with a red X indicate that the gene is missing. The blue circles represent intact genes (CPXV orthologs were used as reference for full-size genes). Blue slices represent ORF fragments that vary in size and number depending on the virus. The blue horizontal triangle in the upper right corner indicates the level of gene disruption in different VACV strains. The least (left) and the most fragmented (right) patterns found in VACV strains are shown. The asterisk indicates the pattern of VACV_DPP17, the only VACV strain with an I4L ortholog truncated in two ORFs.

To integrate the overall results regarding the gene content found on the genomes’ ends of these historical smallpox vaccines, we generated a mandala-shaped Venn diagram (Fig. 4A). The central region of the diagram compares all historical vaccines, but excludes VK05, and reveals that the five vaccines share 24 of those 48 genes. The floating dual-set diagrams surrounding the central mandala show the individual analysis of each ancient vaccine versus HPXV_MNR-76/VK05 (Fig. 4A). The diagrams reveal that VK12 shares 35 of the 43 genes present in HPXV_MNR-76/VK05, whereas MFDV_1902 shares all its 27 genes in the hypervariable region with HPXV_MNR-76/VK05. Regarding the presence of novel genes, VK01, VK08, and VK12 have one unique gene each and VK02 has five unique genes, not present in HPXV_MNR-76/VK05. They all correspond to orthologs of CPXV genes; some are disrupted and annotated as different ORFs, as shown in Fig. 3F.

Because of the high diversity of VACV strains, it was not possible to evaluate all VACV strains individually using the same approach. However, the use of a 50% consensus sequence of several contemporary VACV genomes revealed the same gene set in the hypervariable regions as the MFDV_1902 (data not shown). Together, these results indicate that the high complexity observed in the structure of the VK genome ends is not matched by an increase in the number of novel genes in the telomeric regions. In fact, we observe that all historical vaccines, except VK05, have lost several genes in these regions, compared with the HPXV_MNR-76 genome.

Next, we evaluated the patterns of gene fragmentation in the genomes of the historical vaccines and compared them with those of HPXV_MNR-76 and of different strains of modern VACV. With few exceptions, the central conserved region did not show any significant differences, as expected. On the other hand, the variable regions revealed diverse patterns of gene disruption. For example, several genes in the variable region of the MFDV_1902 and in the modern VACV genomes are more fragmented than their orthologs in VK vaccines and HPXV_MNR-76. Orthologs of HPXV003 (CrmB, encodes a tumor necrosis factor (TNF) alpha receptor-like protein), HPXV004 (encodes an ankyrin domain-containing protein), and HPXV022 (VACV C9L, encodes an antagonist of the type I interferon pathway) (25, 31) are good examples, as shown in Fig. 4B (details in Table S2). Like in CPXV, in VK vaccines, and in HPXV_MNR-76, these accessory genes are intact, while in MFDV_1902 and modern VACV strains, they are disrupted in several small ORFs (Fig. 4B; Table S2). Interestingly though, in the case of the VACV C9L ortholog, only VACV strains of clades I and II have a fragmented gene, whereas strains from the clade III have preserved intact orthologs. For other genes, such as the HPXV039 ortholog (VACV K5L and K6L, which encode monoglyceride lipase), only VK05 and HPXV_MNR-76 have intact or near-intact genes. In HPXV, this ortholog is shorter due to a premature stop codon. MFDV_1902 and all VACV strains have separate K5L and K6L genes fragmented into several ORFs (Fig. 4B; Table S2).

Most interestingly, for other genes, we detected a contrasting pattern of fragmentation. For example, for the VACV I4L ortholog, which is in the conserved region of poxvirus genomes and encodes the large subunit of the viral ribonucleotide reductase (32), the gene is fragmented into three ORFs in all VK viruses and also in MFDV_1902, and into four ORFs in the HPXV_MNR-76 genome. On the other hand, all VACV strains, likewise the CPXV strains, have an intact 2,316 bp gene sequence, except VACV_DPP17, in which the I4L ortholog is split into two ORFs (Fig. 4B; Table S2). This pattern of a given gene being intact in VACV strains and fragmented in the historical vaccines and HPXV_MNR-76 also occurs for other genes, such as C4L and M1L orthologs, among others, but in these cases, only VACV strains of clade III show intact genes (Fig. 4B; Table S2).

### Interesting patterns of SNPs and INDELs found in the central conserved region of the genomes of historical smallpox vaccines

**Fig 5:**
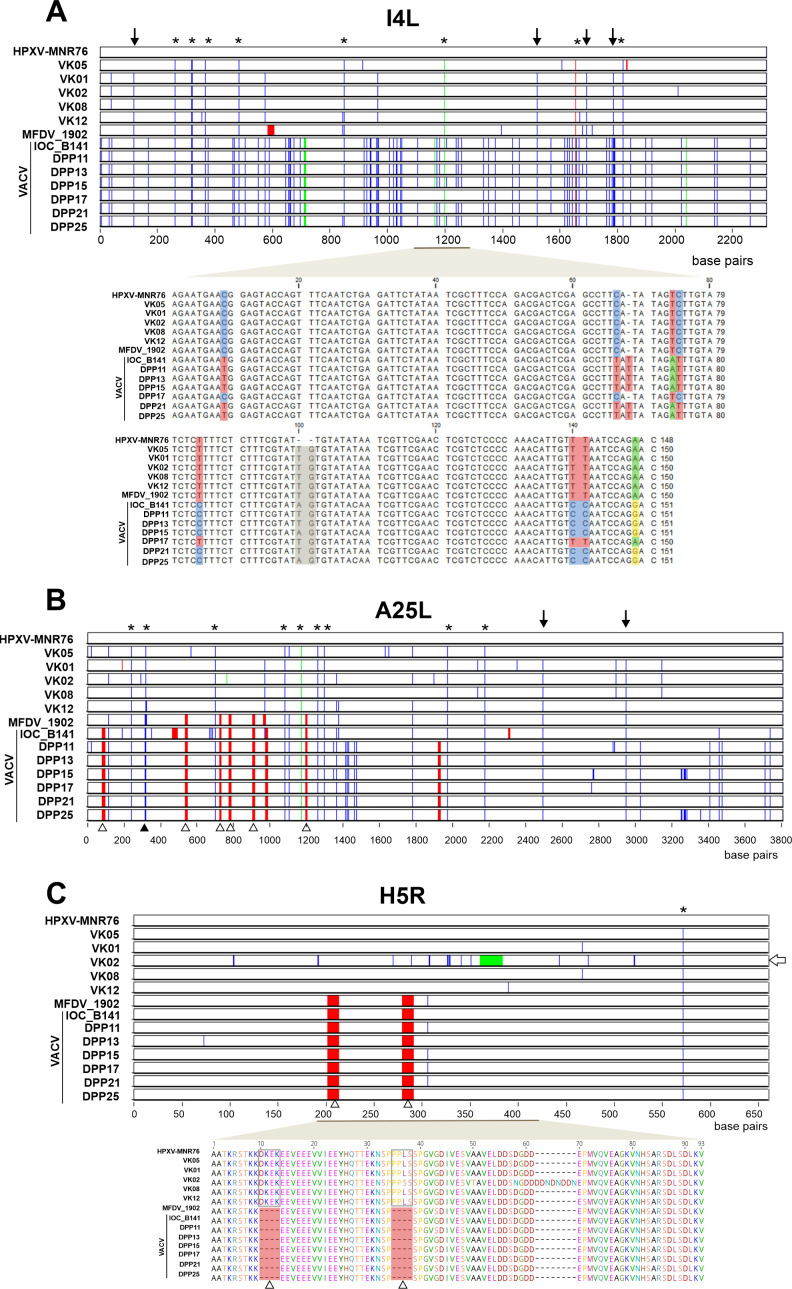
Analysis of polymorphic sites in I4L, A25L, and H5R orthologs in the historical smallpox vaccines. Multialignments of I4L (**A**), A25L (**B**), and H5R orthologs (**C**) were screened for substitutions (blue), deletions (red), and insertions (green) using Base-by-Base; HPXV_MNR-76 was used as reference. Asterisks indicate polymorphic sites present in all viruses but missing in HPXV_MNR-76. Black arrows indicate polymorphic sites present in all viruses but missing in VK05 and HPXV_MNR-76, both having the same pattern. The lower panel in panel A shows details of a stretch of 151 nucleotides of the I4L alignment, highlighting features reported by Qin et al. (17). In the lower panel in panel **C**, details of the predicted amino acid sequence of a 420-nt stretch of sequence from the H5R alignment are shown. Open arrowheads indicate two deletions in MFDV_1902 and in all VACV strains but missing in both the VK vaccines and HPXV_MNR-76.

The central conserved regions of the historical vaccine genomes share >99.5% similarity with HPXV_MNR-76 (Fig. 1C). Yet, several SNPs and INDELs have accumulated along the genomes, as shown in Fig. 1B. The changes seen at some loci are particularly interesting. For example, the I4L ortholog in VACV strains accumulates more SNPs and INDELs than the historical vaccine orthologs, compared to HPXV_MNR-76 (Fig. 5A, top panel). Some changes were shared among all VK viruses, the MFDV_1902, and the VACV strains (Fig. 5A, asterisks), whereas other variants were not shared with VK05, which has the same sequence as HPXV_MNR-76 in some positions (Fig. 5A, arrow).

Qin et al. (17) reported the presence of unique variants in the HPXV_MNR-76 I4L ortholog that were missing in all VACV genomes, except the Dryvax clone DPP17 (17). These variants are highlighted in the alignment shown in the lower panel of Fig. 5A. We noticed that all VK viruses and the MFDV_1902 shared the same variants with HPXV_MNR-76, except for a 2-nt deletion, which was unique to the HPXV_MNR-76 genome.

Another interesting locus is the VACV A25L ortholog, which encodes a truncated version of the cowpox A-type inclusion protein (32). All HPXV-like viruses and VACV strains share a similar pattern of gene disruption (data not shown). As observed for the I4L ortholog, several SNPs in the A25L gene are shared by all viruses, except HPXV_MNR-76 (Fig. 5B, asterisks), while for other SNPs, VK05 and HPXV_MNR-76 shared the same pattern but differed from that seen in the other historical vaccines and VACV strains (Fig. 5B, arrows). Interestingly, we noticed that VACV strains and MFDV_1902 share an SNP and several deletions in the A25L ortholog not found in the other historical vaccines (Fig. 5B, black and white arrowheads, respectively).

A similar scenario occurred with the VACV H5R ortholog, which encodes the viral late transcription factor 4 (VLTF-4) (32). The H5R orthologs of all VACV strains have two 12-nt deletions also shared with its ortholog in MFDV_1902, but not with VK viruses or HPXV_MNR-76 (Fig. 5C, white arrowheads). Other genes in the conserved region, e.g., F7L and A31R orthologs, also have deletions shared with MFDV_1902 and VACV strains, but not with the VK viruses or the HPXV_MNR-76 (data not shown). The finding of such “VACV islands” inserted into regions of the MFDV_1902 genome that are otherwise highly similar to HPXV is in line with the finding that MFDV_1902 is the HPXV-based vaccine that shares the highest identity score with VACV (Fig. 1C).

### VK02 vaccine is a recombinant virus carrying sequences of variola virus genes

Of all the ancient vaccines analyzed here, VK02 has the most divergent genome (Fig. 1C), which agrees with the distinct patterns of SNPs and INDEL observed in its genome (Fig. 1B), and it is quite noticeable in some genes, such as the H5R ortholog (Fig. 5C, white arrow).

**Fig 6:**
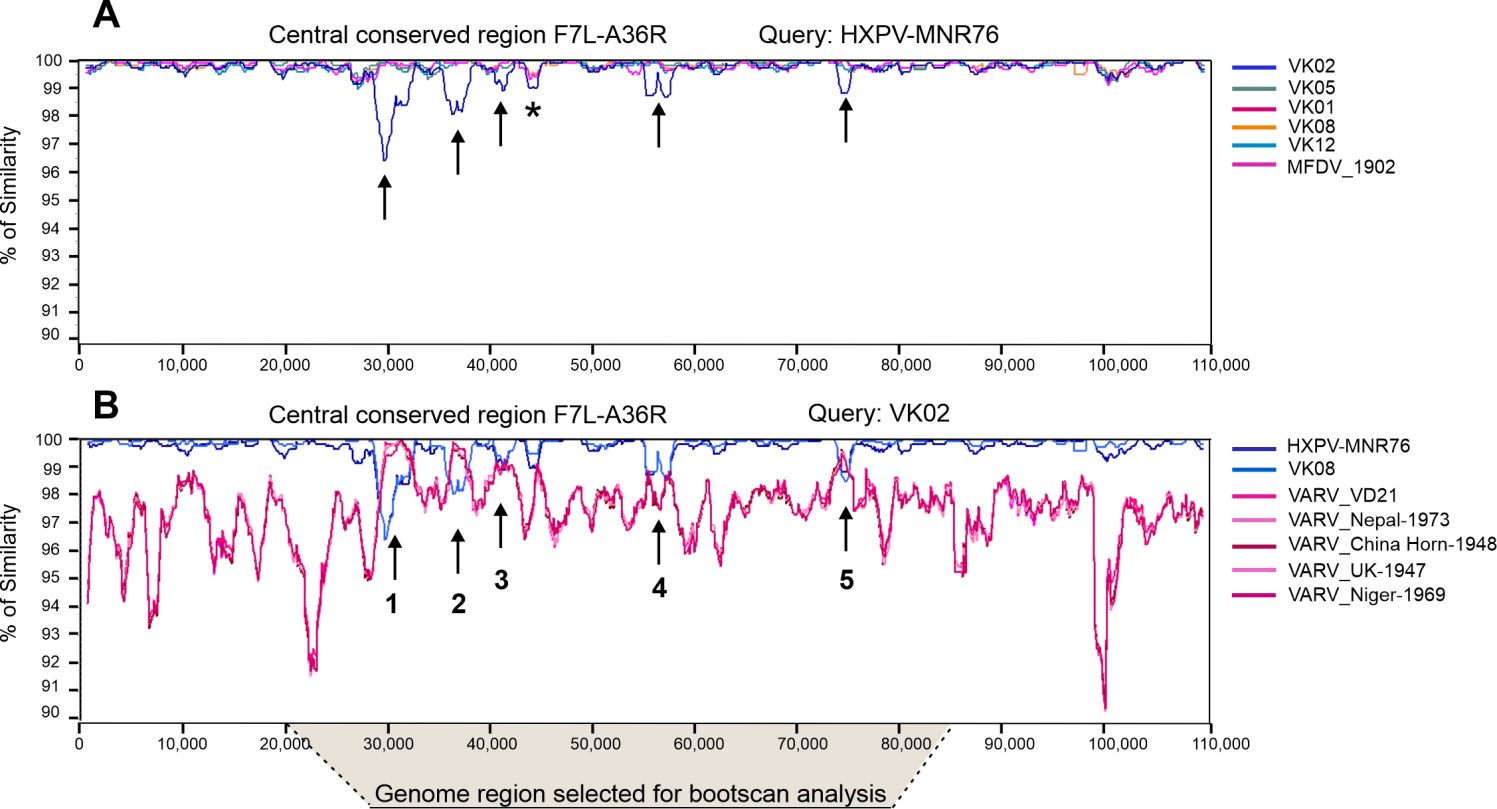
Similarity profile of the central conserved region of the genomes of historical smallpox vaccines. (**A**) The multialignment of an expanded central conserved region (spanning from the F7L to A36R orthologs) of the six historical smallpox vaccines and HPXV_MNR-76 was scanned for percent similarity using Simplot, using the HPXV_MNR-76 sequence as query, window size 1,200, step size 120. Black arrows indicate the regions of least similarity between VK02 and all other genomes. The asterisk indicates a region of least similarity of all vaccines relative to HPXV_MNR-76. (**B**) The multialignment included five variola virus strains and removed all historical vaccines, except VK02 and VK08. Similar parameters were chosen, but VK02 was selected as the query. The black arrows indicate the regions of least similarity between VK02 and HPXV and VK08, and of high similarity between VK02 and variola virus. Numbers refer to the regions chosen for the Bootscan analysis, from 20 kb to 85 kb of the alignment.

To further investigate the extent of the VK02 genome divergence, we used Simplot to scan the central conserved region and generate a similarity plot in which the HPXV_MNR-76 sequence was used as the query (Fig. 6A). The analysis spanned an extended conserved region of approximately 110,000 bp from F7L to A36R orthologs. As shown in Fig. 1B, the central regions of all historical vaccines are highly similar to that of HPXV_MNR-76. However, VK02 has six regions that bear the least similarity to HPXV_MNR-76, five of which share little similarity to HPXV, and all viruses analyzed (Fig. 6A, black arrows).

These five regions of lower similarity in the VK02 genome were used as queries in Blastn searches in the National Center for Biotechnology Information (NCBI) database. Surprisingly, the best 50 hits were sequences from VARV. The hits varied from sharing 99.72% to 97.35% similarity with different VARV strains. The reassembled sequence of the ancient strain VD21 (33) isolated from a 17th century mummy from Lithuania (34) was the most similar hit for three of the five regions tested. For the other two regions, VARV strain Niger-1969 was the best match or VARV strain Niger-1969 along with nine other strains, but not VD21, were the best hits (Table S3).

Next, we added the central conserved region of five top-hit VARV sequences to the alignment. Simplot was then used to scan the alignment and generate a similarity plot, this time using the VK02 sequence as the query. For the sake of clarity, we removed all historical vaccine genomes, except VK08, which share the highest similarity score with VK02 in the central genome (Fig. 1C). The plot shown in Fig. 6B reveals that VARV sequences share an overall lower similarity ratio with the VK02 genome, when compared with the >99% average similarity rate that VK02 shares with the genomes of VK08 and HPXV_MNR-76. However, the five specific regions of lower similarity shared between VK02 and both VK08 and HPXV_MNR76 overlap with peaks of higher similarity shared between VARV sequences and VK02 (Fig. 6B, black arrows). Similar results were obtained when we replaced VK08 with VK05 in the analysis or when we used a 50% consensus sequence of VK01, VK05, VK08, and VK12 (data not shown). These findings suggest the occurrence of recombination events involving VARV in the evolutionary path of VK02 virus.

**Fig 7:**
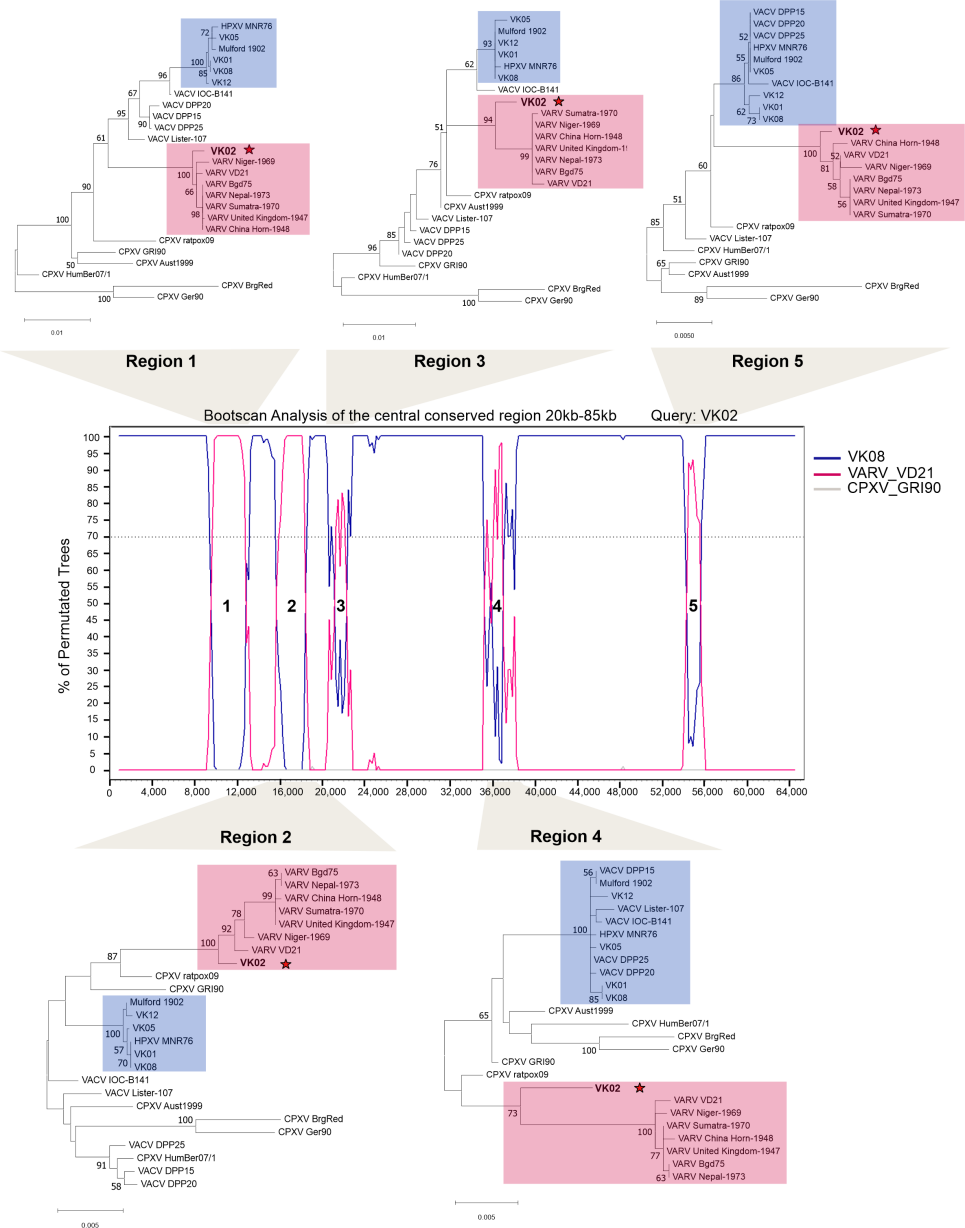
Recombinant regions in the central conserved region of the VK02 genome. The region between 20 kb and 85 kb of the multialignment shown in Fig. 6 was cut out and analyzed by Bootscan, using VK02 as query, window size 1,700 and step size 200. Five putative recombinant regions with VARV were identified. VK02 sequences between recombination breakpoints were extracted and realigned with several orthopoxviruses for phylogenetic analysis using either maximum likelihood or neighbor-joining models, as described. Blue boxes indicate the HPXV and HPXV-related cluster, and pink boxes indicate the VARV cluster. The position of VK02 in the trees is indicated by a red star. The numbers indicate bootstrap support of 1,000 replicates (>50% is shown). The scale bar indicates the number of substitutions per site.

To confirm our hypothesis and locate the specific positions of the recombination breakpoints, we carried out Bootscan analyses (Fig. 7) of a nested region of 65,000 bp containing all five putative recombination regions indicated in Fig. 6B. The VK02 sequence was used as the query, VK08 and VARV_VD21 (reassembled genome) (33) were chosen as surrogates for potential parental donors, whereas CPXV_GRI-90 was used as a non-donor sequence.

The Bootscan profile detected five recombination events with VARV sequences within the central conserved region of VK02 (Fig. 7). To validate these observations, VK02 sequences within the breakpoints of each event were aligned with several orthopoxviruses and used for phylogenetic inference. The trees for each region confirmed that VK02 grouped with VARV strains, mapping at the root of the VARV cluster (Fig. 7, pink boxes) and not within the HPXV cluster like the other historical smallpox vaccines did (Fig. 7, blue boxes).

Although the VK02 position was supported by high bootstrap values for most regions (>90), regions 3 and 4 were not well resolved in the Bootscan profile. Therefore, for better accuracy, the 65 kb alignment was broken down into five regions, each including one of the five recombination events, which were analyzed again by Bootscan with fine adjustments in the sliding window size and window step size. For this new analysis, VK08, reassembled VARV_VD21, (33) or VARV_Niger-1969 were chosen as surrogates for potential parental donors, according to the best VARV hit indicated for each region in Table S3. CPXV_GRI-90 was used as a non-donor sequence. Regions 1 to 4 revealed more than one recombination event with VARV, and these subregions were designated by alphabetical letters (Fig. S3 to S6). The new analysis of region 5 did not show any recombination subregion, revealing the same profile shown in Fig. 7 (data not shown). Each recombination subregion in regions 1 to 4 was used for phylogenetic inference. The trees validated our hypothesis and show that VK02 grouped within the VARV cluster or mapped immediately before the divergence point of the VARV group, sometimes with similar topology as the 17th-century VARV_VD21 or VARV_Niger-1969. For most inferences, the assignment of VK02 to the VARV group was supported by high bootstrap values (>80) and only a few had less than 70, but were still above 60 (Fig. S3 to S6).

**Fig 8:**
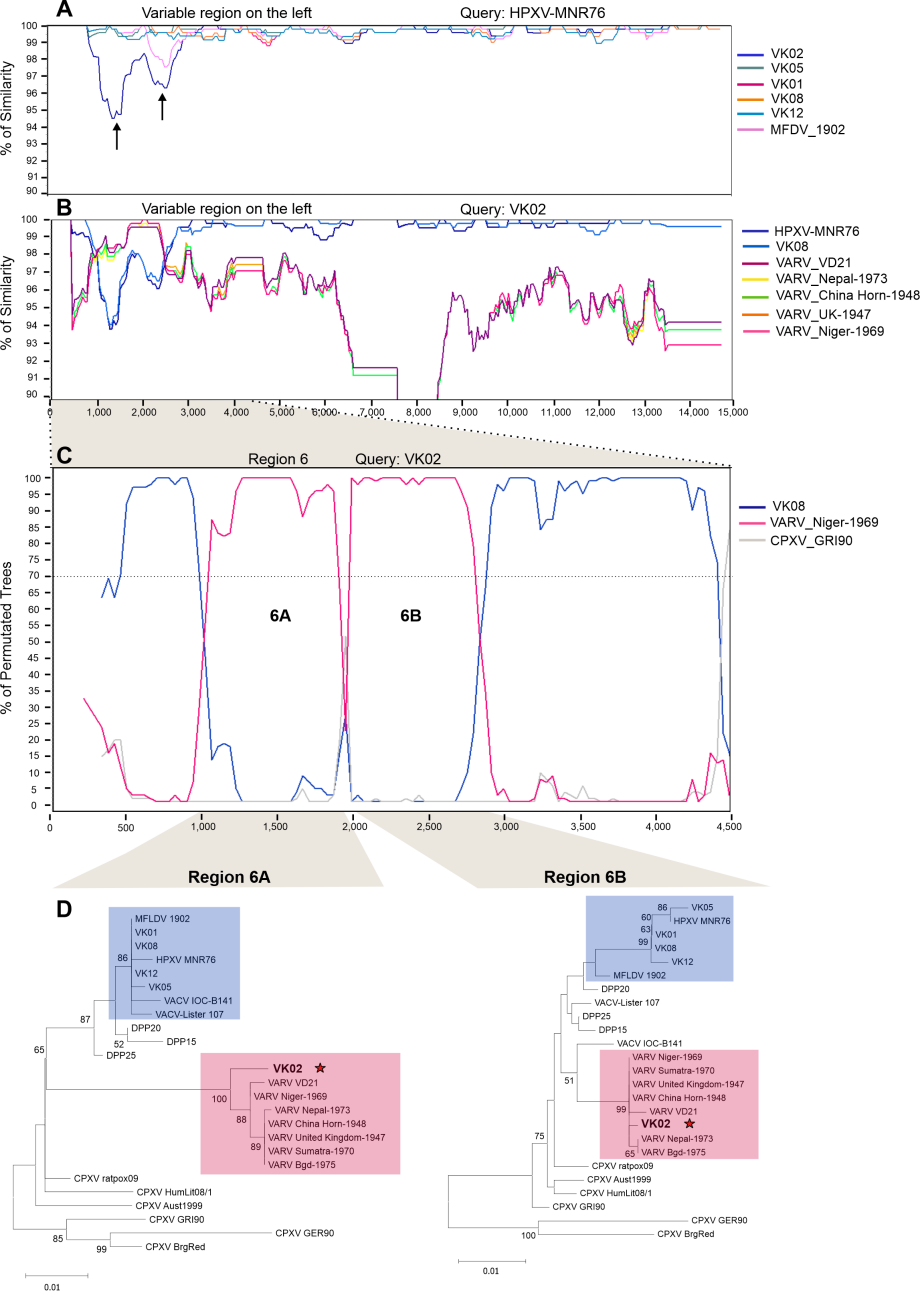
Recombination in the variable region at the left end of the VK02 genome. (**A**) The multialignment of the variable region at the left end of the genomes of the six historical smallpox vaccines and HPXV_MNR-76 was scanned for percent similarity using Simplot, using the HPXV_MNR-76 sequence as query, window size 500, step size 50. Black arrows indicate the two regions of least similarity between VK02 and all other sequences, including HPXV_MNR-76. In one of the regions, in addition to VK02, MFDV_1902 also shares low similarity with the other viruses. (**B**) The multialignment included five variola virus strains and excluded all historical vaccines, except VK02 and VK08. Simplot was used with similar parameters, using VK02 as the query. Black arrows indicate regions 6A and 6B of least similarity between VK02 and both HPXV and VK08, and of highest similarity between VK02 and variola virus. (**C**) The first 4.5 kb of the multialignment shown in panel B was analyzed by Bootscan, using VK02 as query, window size 300, and step size 30. The two putative recombinant regions with VARV 6A and 6B are indicated. The VK02 sequences between recombination breakpoints were extracted and realigned with several orthopoxviruses for phylogenetic analysis using either maximum likelihood or neighbor-joining models. Blue boxes indicate the HPXV and HPXV-related cluster, and pink boxes indicate the VARV cluster. The position of VK02 in the trees is indicated by a red star. The numbers indicate the bootstrap support of 1,000 replicates (>50% is shown). The scale bar indicates the number of substitutions per site.

The detection of recombinant regions in the central part of VK02 genome led us to investigate whether other recombination events may have occurred in both variable regions. Simplot analyses of the left and right ends were carried out using HPXV_MNR-76 as the query. There was no indication that recombination events have occurred at the right end, but two regions at the left end revealed lower similarity scores when VK02 was compared with HPXV-MNR-76 and other historical vaccines (Fig. 8A, black arrows). In one of the regions, MFDV_1902 also had a profile similar to that of VK02, which was investigated separately.

The VK02 sequence within the two regions was used to run Blastn searches. Again, VARV strains appeared as the top 50 hits (Table S3), but not MFDV_1902. The VARV sequences were then included in the alignment, and we carried out a new analysis in Simplot, now using VK02 as the query. The similarity plot confirmed the greatest similarity between the VARV and VK02 sequences in the labeled regions 6A and 6B, suggesting that recombinant events with VARV occurred in the variable region at the left end of the VK02 genome (Fig. 8B).

The initial 4.5 kb of the alignment that includes the 6A and 6B regions was Bootscanned using VK02 as the query, plus VK08 and VARV_Niger-1969 (the best match according to Table S3) as surrogate sequences for potential parental donors (Fig. 8C). The Bootscan profile indicates the occurrence of two recombinant events involving VARV that were validated by phylogenetic analysis of the sequences between breakpoints, supporting the idea that recombination events also occurred in the variable region (Fig. 8D).

To rule out genome misassemble, the raw reads were remapped against the VK02 contig to confirm that there were no mapping inconsistencies, and to inspect the read density in the six recombination regions, particularly at their boundaries (Fig. S7). We also tried to map unmapped reads against the VARV genome to no avail, indicating that there were no isolated VARV DNA molecules mixed in the VK02 sample (data not shown). Therefore, we did detect bona fide copies of VARV sequences recombined into the VK02 genome.

Regarding the stretch of MFDV_1902 sequence sharing lower similarity with HPXV_MNR-76 in region 6B, Blastn searches of this region retrieved different VACV sequences as top hits, instead of VARV. This same strategy was performed to include VACV sequences in the Simplot analysis, followed by Bootscan analysis and phylogenetic inference of the sequences between breakpoints. The resulting tree had no statistical support to validate the inclusion of MFDV_1902 in any VACV cluster, although it did not group with the historical vaccines either (Fig. S8).

**Fig 9:**
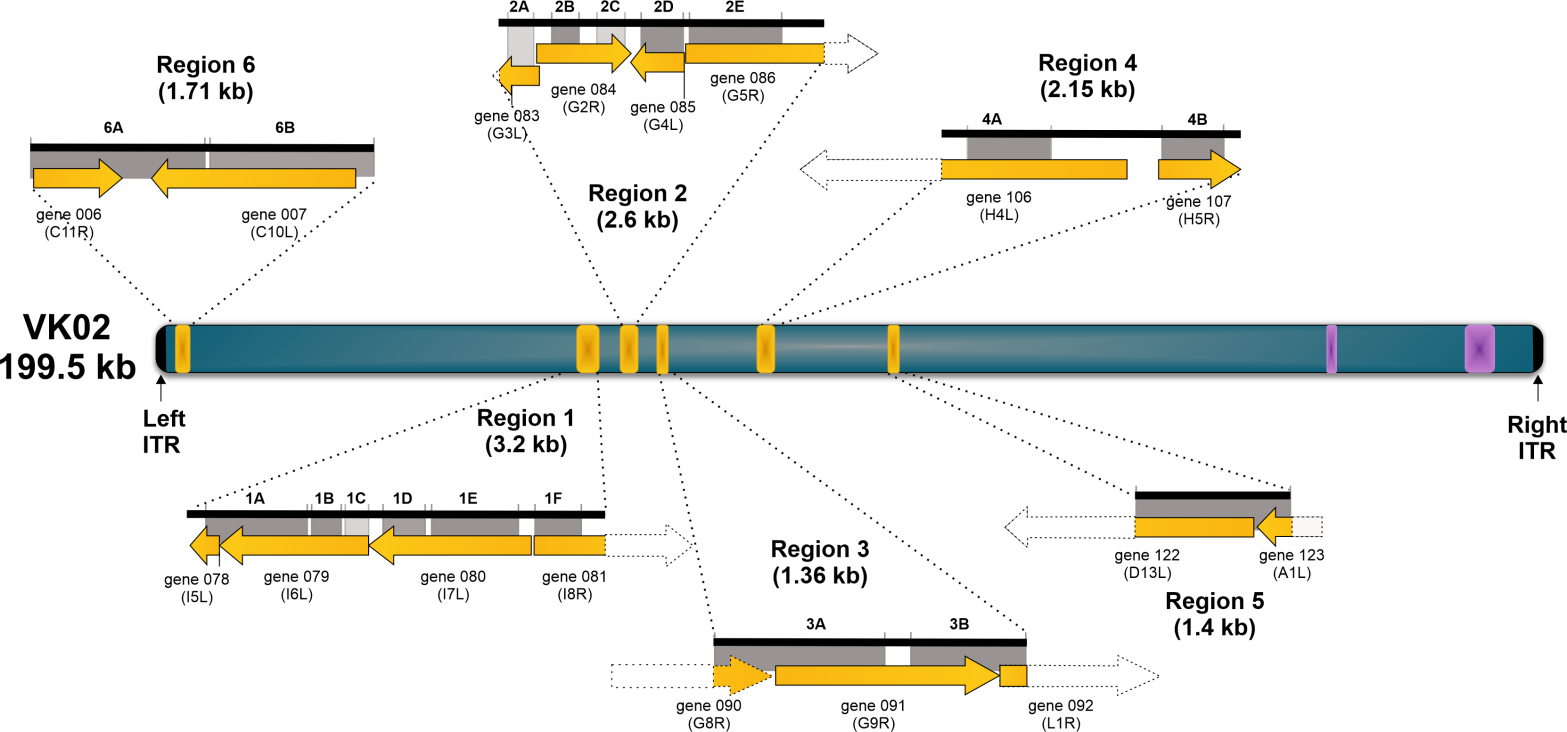
Schematic diagram of the VK02 genome. The yellow boxes represent the six VARV recombination regions in the VK02 genome that were detected by Bootscan analysis and supported by phylogenetic inference. Each region is zoomed in to show the genes involved in the recombination events (yellow arrows). The dotted lines on the arrows represent parts of the VK02 genes that are outside the area of recombination with VARV. Recombination subregions are indicated above the black lines, and gray areas indicate the bootstrap support by the phylogenetic analysis. Light gray, 60% < 70%; dark gray, >70%. Purple boxes indicate regions with CPXV genes in the VK02 genome.

Figure 9 shows a scheme of the VK02 genome and highlights the genes and regions involved in the recombination events with VARV. The recombined regions include the partial sequence of several essential genes involved in virus gene expression and replication processes, such as the orthologs of I6L, I7L, G4R, G5R, and G9R, and the full sequence of essential genes such as the H5R ortholog. Important accessory genes have also suffered full and partial recombination, such as the orthologs of C11R and C10L, respectively. A partial alignment of C11R orthologs (region 6A) is shown as an example in Fig. S9, which also shows the location of the recombination breakpoints in the VK02 genome.

## DISCUSSION

The numerous gaps in the history of the smallpox vaccine overlap with missing data on the evolutionary path of VACV and the closely related HPXV (2, 5, 26, 35, 36). Our study adds information to the evolutionary history of VACV and HPXV-based smallpox vaccines by analyzing in detail the genomes of six historical smallpox vaccines used in the United States from the mid-19th century to early 20th century.

Except for VK05, which is a bona fide HPXV, the other five VK vaccines partially lost the HPXV-specific genomic features on the genome ends, while MFDV_1902 lost them completely, similar to what may have occurred to contemporary VACVs. The VK01 and VK12 vaccines have complex genomes with large stretches of duplicated accessory genes inserted into their variable left terminal regions, increasing their ITR size and gene dosage. Interestingly, in MFDV_1902 and modern VACVs, the stretch of duplicated genes is smaller and only five of these genes are still found.

The VK02 vaccine is quite interesting and represents a recombinant virus carrying both partial and full genes of variola virus in its genome. The interspecies homologous recombination events targeted mostly the conserved region, e.g., the locus of the H5R ortholog, which encodes the VLTF-4 and the G9L ortholog, which encodes one component of the entry-fusion complex (32). Furthermore, we also detected recombination events in the variable region at the left end of the genome, in a region where two important accessory genes are located, namely the orthologs of C11R, which encodes the virus epidermal growth factor, and C10L, which encodes the interleukin-1 receptor antagonist and the DNA-dependent protein kinase(PK) binding protein (25, 31).

Recombination plays an important role in driving poxvirus evolution, particularly when it involves the recombination of intermediate-sized fragments and genome microregions (35 – 39). These events lead to mosaic genomes that display a “patchy pattern” that, in the case of smallpox vaccines, can reveal “scars” from past recombination events, such as molecular fossils (15, 17, 35) or footprints from possibly recent events, such as the “vaccinia islands” observed in different regions of the MFDV_1902 genome. It is reasonable to assume that the events observed for the VK02 vaccine may have impacted its virulence and reactogenicity. However, unfortunately, it is not possible to investigate this hypothesis, as no infectious virus particles were recovered from the original vaccine samples (20).

According to Duggan and colleagues, the VK02 DNA was isolated from crusts present in vaccination kits from the Mutter Museum collection (20). However, the date and further information on the material are unavailable, which limits the effort to understand the origin of this recombinant smallpox vaccine. Despite this missing information, history accounts for numerous practices that offered potential opportunities to mix smallpox-derived material with the smallpox vaccine. For example, some vaccinators mixed smallpox scabs, or pus, with the smallpox vaccine, hoping to potentialize vaccine (6). Variolation of cows with smallpox pus and subsequent inoculation in individuals, in an attempt to produce smallpox vaccines, was also widely tested in Europe and the United States during the 19th century (6 – 8, 40, 41). It was also a common practice to variolate individuals with smallpox pus a few days after vaccination as a way of testing the vaccine’s efficacy. The vaccine pustule was then passed arm-to-arm to other individuals, usually using the same lancet in both procedures (41).

An aspect that needs further discussion is whether these historical smallpox vaccines truly represent the ancestors of modern VACV strains. Phylogenetic analyses, shown here and in previous studies, place the HPXV and HPXV-related vaccines as a well-supported group within the VACV lineage (15 – 17, 20). This close relationship with modern VACV strains results from the high scores of nucleotide similarity in the conserved region of the genome, which is usually used as the basis for phylogenetic inferences. However, this genetic relatedness is only a partial view of the matter. Other features that are important for our understanding of VACV lineage ancestry are not considered in phylogenetic inferences, as many unsampled intermediate players are absent, and recombination or introgressive events that affect microregions are eclipsed in the analysis of large genomes. The method also does not consider relevant characteristics for understanding the evolutionary path of the VACV lineage, such as gene pool, gene integrity, and especially the structure of the genome (25, 27, 36). Regarding these structural features, we and others have noticed that HPXV and CPXV are similar in their genome architecture, particularly in their terminal regions, with a similar gene set (14, 25, 30, 42). Therefore, a broader approach to investigate the origins of VACV and HPXV-like viruses would need to consider both nucleotide similarity and the structural genomic features and gene set mentioned above. As CPXV is considered to be most similar to what was once the ancestor of the genus *Orthopoxvirus* (26 – 30), we then believe that members of the HPXV subclade would, in fact, occupy an earlier position in the VACV lineage.

The analysis of the fragmentation pattern of some genes strongly suggests that, despite this putative ancestral position, none of the historical American smallpox vaccines analyzed in this work could be considered the progenitor (direct ancestor) of contemporary VACV strains. The fragmentation pattern of the I4L ortholog is a typical example. Like CPXV, all modern VACV strains have an intact I4L ortholog, except for the Dryvax clone DPP17. However, in all historical smallpox vaccines analyzed in this study, the I4L ortholog is fragmented. These findings not only support previous analysis of the HPXV_MNR-76 genome that excluded its role as a progenitor virus of modern VACV (14), but they also apply to the six historical vaccines.

Our results support not only the recombinogenic nature of the orthopoxviruses, as discussed, but also the current theories of reductive evolution. Such theories advocate that during orthopoxvirus evolution and adaptation to new niches/hosts, different selective pressures on different genes may have led to an increasing build-up of SNPs and INDELs, generating early stop codons, gene truncation/fragmentation, and ultimately resulting in gene loss and shorter genomes (25 – 27, 29, 36). Our analysis also highlights a role for the gene duplication/transposition that occurred in the variable ends, which led to gene loss and the contraction and expansion of the ITRs, a phenomenon that Hatcher and coworkers (27) previously investigated with other orthopoxviruses (27). Different models of intragenomic and intergenomic nonhomologous recombination events have been proposed to explain the contraction and expansion of the ITRs in clones of the Dryvax vaccine and could certainly explain the events of gene loss and gene duplication/transposition described in this study (17, 26, 43).

**Fig 10:**
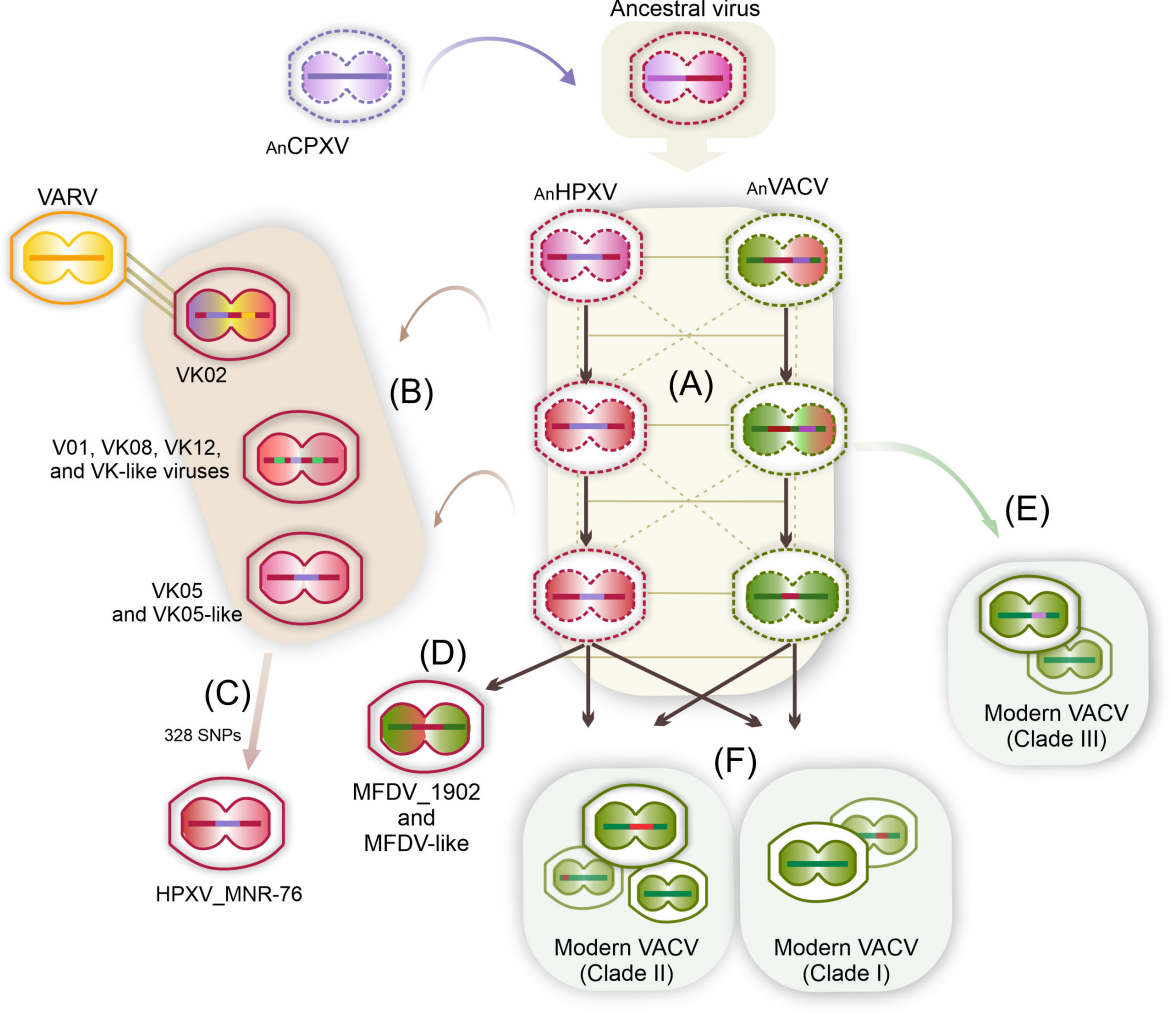
Our model for the evolution of HPXV-related smallpox vaccines and modern VACV. Unsampled ancestral viruses are indicated by the dotted structure. The ancestral virus would have mixed features of VK05/HPXV and CPXV, i.e., similar genome architecture and intact genes like CPXV, but greater nucleotide identity with VK05. (**A**) After several passages of the putative ancestral virus, two lineages of the oldest smallpox vaccines, the ancestral HPXV (AnHPXV) and the ancestral VACV (AnVACV), were generated. Coevolution and, at some point, co-passage of these two lineages favored the occurrence of genetic flow (thin horizontal lines) and introgressive events with backcross hybridization with parental viruses (vertical and crossed dotted lines). (**B**) Few or multiple stochastic sampling events during AnHPXV lineage passage would lead to the generation of VK vaccines accompanied by some level of gene fragmentation (e.g., I4L). VK05 retained the characteristic HPXV genes, whereas other VK viruses partially lost them but retained some CPXV genes. Co-culture with VARV favored recombination events that generated VK02. (**C**) The evolution of VK05-like viruses included the build-up of SNPs generating what is known as HPXV_MNR-76. (**D**) Later, during the continuous passage of AnHPXV, several genes were lost, becoming what is now represented by the MFDV_1902 vaccine. (**E**) Stochastic sampling of AnVACV during early passage events generated VACV clade III, which retained several intact genes (e.g., I4L, M1L, and C4L) and some fragmented cowpox genes (Lister-related strains). (**F**) After further passages and co-cultivation of both ancestral lineages, some gene fragmentation (C4L and M1L, for examples) occurred while other genes remained intact (for example, I4L). Different sampling events and subsequent geographically separated propagation would lead to the separation of VACV strains into sister clades I and II. Clade II likely received a greater contribution from HPXV sequences than clade I.

Based on our findings and on current theories of *Orthopoxvirus* evolution, we propose a model for the evolution of the HPXV and VACV lineages (Fig. 10). We suggest that a putative ancestral virus of smallpox vaccines would have the same genomic architecture as CPXV/VK05/HPXV, a complete set of intact genes similar to CPXV but with a superior nucleotide identity to VK05. Interestingly, cowpox viruses form a polyphyletic group that is quite divergent in nucleotide identity among the five clades. However, they show strong relatedness in genomic structure, gene content, and gene integrity, which has led to intense debates over whether they should still be collectively called cowpox viruses (28, 30, 44, 45). For the sake of discussion, we ask whether there was in the past a CPXV clade that was even closer to the VACV lineage than the current VACV-like clade. This CPXV clade would have the characteristics proposed above in order to be the ancestral virus.

Passages of this putative ancestral virus possibly in different hosts may have generated the oldest smallpox vaccines that would be grouped into two lineages, the ancestral HPXV (AnHPXV) and the ancestral VACV (AnVACV). We suggest that these two lineages co-evolved and, at some point, were probably co-passaged, which favored the occurrence of genetic flow and introgressive events even with backcross hybridization with parental viruses (Fig. 10A). This process probably promoted the generation of genetically diverse genomes in both lineages.

Few or multiple stochastic sampling events during the passage of the AnHPXV lineage likely led to the generation of the VK vaccines (Fig. 10B). These viruses may have been co-cultured, and possibly backcrossed with their parental viruses, which would favor the occurrence of several changes observed in their genomes. All VK vaccines, except VK05, preserved some cowpox genes likely present in early AnHPXVs. VK01, VK08, and VK12 partially lost genes from the 10.7 kb insertion, while VK02 suffered massive gene loss and contraction of ITRs. On the other hand, VK08 and VK12 gained long stretches of genes as a result of duplication/transposition events, and the VK02 genome gained VARV sequences by homologous recombination.

VK05 no longer has the CPXV genes, but retains all genes in the 10.7 kb and 5.5 kb insertions similarly to HPXV_MNR-76. However, during evolution and adaptation to new niches, some genes accumulated SNPs and increased the fragmentation pattern, as observed in the HPXV_MNR-76 genome (Fig. 10C). Thus, we propose that this natural field isolate of HPXV represents a dead-end result of this evolution path.

At some late point during the continuous passage of the AnHPXV lineage, several genes were lost, becoming what is now represented by the MFDV_1902 vaccine (Fig. 10D). All CPXV-specific genes and the 10.7 kb and 5.5 kb insertions were completely lost, and most of the genes in the duplicated/transposed sequences at the left end of VK01/VK12 were also lost, retaining only five accessory genes, similar to several contemporary VACV strains.

Unlike AnHPXV viruses, the AnVACV lineage preserved several genes intact, such as the I4L, C4L, and M1L orthologs. During the early passage events of the AnVACV lineage, stochastic sampling also occurred, which may have originated the VACV clade III (Fig. 10E). At this point in passage history, AnVACVs still retained some CPXV genes at the right end of the genome, which were retained, albeit fragmented, in some clade III VACV strains. An early split of this VACV clade is in line with its greater genetic distance to HPXV than members of clade I and II, and was also proposed by Molteni et al. (35). However, we cannot exclude geographic bias for the historical vaccines analyzed in this study, as discussed below.

After clade III split, further passage of the AnVACV lineage led to fragmentation of some genes such as C4L and M1L, but others, such as I4L, remained intact, unlike the AnHPXV lineage. Further co-passage of both ancestral lineages favored additional gene flow and introgressive events. At some point, different sampling events, perhaps facilitated by different geographic locations where smallpox vaccines were produced, led to the separation of VACV strains into sister clades I and II (Fig. 10F). Based on the genomes available so far, clade II probably received a greater contribution of HPXV sequences than clade I.

One limitation of the proposed model is that all vaccines analyzed in this study were produced or used in the United States, which restricts our discussion and makes us wonder whether they truly represent historical smallpox vaccines used worldwide in the 19th century. On the other hand, in the mid-19th century, European countries were the main distributors of smallpox vaccine seeds to the world (46). Therefore, it is reasonable to assume that the genetic makeup of those historical American vaccines may carry features of smallpox vaccines manufactured in Europe at that time, but further sequencing and analysis of vaccine genomes from different parts of the world, particularly Europe, is needed.

Unfortunately, we were unable to estimate the divergence time of the members of the HPXV subclade due to imprecise dates of the vaccines, reduced number of samples, serial passages in animals for vaccine production, and recombination events that hinder molecular-dating analysis. However, we wonder why the HPXV subclade went extinct (most likely) and what forces led to the disappearance of horsepox-related vaccines. These answers may be available someday if we obtain more information on the genomes of HPXV-based vaccines from different countries and on the events that occurred before HPXV-related vaccines were replaced by contemporary vaccinia viruses (smallpox vaccines), in the first half of the 20th century. Different selective pressures may have impacted this switch, such as vaccine reactogenicity, virus yield in calves, and vaccine effectiveness.

A better understanding of the evolutionary path of the potentially extinct HPXV subclade will increase our knowledge about the paths that led to the consolidation of current smallpox vaccines. This is particularly important today, when the potential resumption of smallpox vaccine production to be used against mpox is widely discussed and improved vaccines are desirable.

## Data Availability

The genome sequences analyzed in this work are available in GenBank. All sequence records are listed in Table S1.

## SUPPLEMENTAL MATERIAL

Supplemental Figures and Tables mbio.01887-23-s0001.pdf Figures S1 to S9; Tables S1 to S3.

ASM does not own the copyrights to Supplemental Material that may be linked to, or accessed through, an article. The authors have granted ASM a non-exclusive, world-wide license to publish the Supplemental Material files. Please contact the corresponding author directly for reuse.
