# Supplementary material for: Gene duplication, gene loss, and recombination events with variola virus shaped the complex evolutionary path of historical American horsepox-based smallpox vaccines
Source: mBio. 2023 Sep 20;14(5):e01887-23. doi: 10.1128/mbio.01887-23 (PMC10653919; doi:10.1128/mbio.01887-23)
Supplement: Supplemental Figures and Tables — Figures S1 to S9; Tables S1 to S3. [file mbio.01887-23-s0001.pdf]

Supplemental material

Gene duplication, gene loss, and recombination events with variola virus shaped the complex evolutionary path of historical American horsepox-based smallpox vaccines

Aline R V Souza[1#], Annika Brinkmann[2], José Esparza[3], Andreas Nitsche[2], Clarissa R Damaso[1*]

[1]Instituto de Biofísica Carlos Chagas Filho, Universidade Federal do Rio de Janeiro, Rio de Janeiro, Brazil. [2]Centre for Biological Threats and Special Pathogens 1 – Highly Pathogenic Viruses & German Consultant Laboratory for Poxviruses & WHO Collaborating Centre for Emerging Infections and Biological Threats, Robert Koch Institute, Berlin, Germany. [3]Institute of Human Virology, University of Maryland School of Medicine, Baltimore, MD, USA

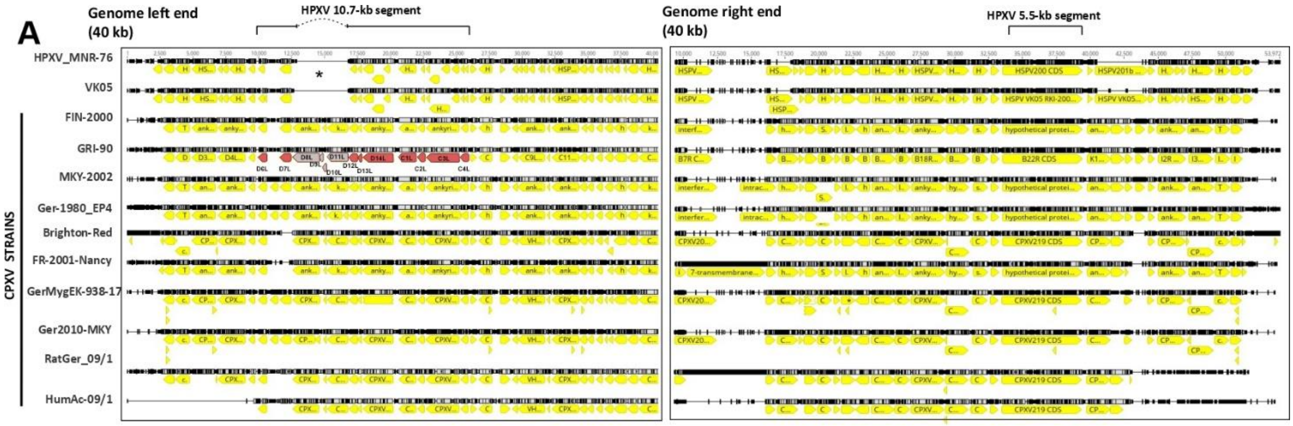

B Nucleotide indentity of HPXV_MNR-76 and VK05 genomes with CPXV strains from the 5 clades.

CPXV CLADE [a]	VIRUS	WGS	CONSERVED REGION
		% IDENTITY (Relative to HPXV_MNR-76)	
	HPXV_MNR-76	100.00	100.00
	VK05	99.69	99.80
VACV-like (Clade 1)	CPXV_FIN-2000	92.87	98.00
	CPXV_GRI-90	92.28	97.99
CPXV-like 1 (Clade 3)	CPXV_MKY-2002	90.94	97.19
	CPXV_Ger-1980_EP4	90.54	97.19
CPXV-like 2 (Clade 4)	CPXV_Brighton-Red	89.03	96.60
	CPXV_France-2001-Nancy	87.69	96.61
CPXV-like 3 (Clade 5)	CPXV_GerMygEK-938-17	89.60	96.58
	CPXV_Ger2010-MKY	89.57	96.62
VARV-like (Clade 2)	CPXV_RatGer_09/1	86.68	97.76
	CPXV_HumAc-09/1	84.10	97.76

[a] Clade designation followed Franke et al (2017) and Senkenvitch et al. (2022), in parenthesis.
WGS: whole-genome sequence.

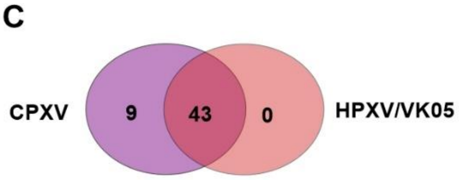

Figure S1. Comparison of genomic structure, percent identity, and gene content among CPXV strains, HPXV_MNR-76, and VK05. A) Alignment of the variable regions of the genomes of CPXV strains from the five clades, HPXV_MNR-76 and VK05. The ORF map highlights the similarity between the CPXV strains, HPXV_MNR-76, and VK05 at the variable ends. The HPXV and VK05 ORFs in the 10.7 kb region (left panel) and in the 5.5 kb region (right panel), which are absent in all VACV strains, are indicated above the figures. The nine CPXV orthologs found in the corresponding 10.7 kb region are highlighted in red only in the CPXV_GRI-90 genome, but they are also seen in the other CPXV strains, according to the alignment. The asterisk indicates the 4.7 kb region found in CPXV strains but absent in HPXV_MNR-76 and VK05. CPXV genes found in this region are highlighted in gray in CPXV_GRI-90. **B)** Pairwise comparison of the percent of shared identity between HPXV_MNR-76, VK05, and two CPXV strains from each of the five clades. **C)** Venn diagram showing the number of genes in the hypervariable regions that are shared by CPXV and HPXV/VK05.

VK01- read mapping - duplication/translocation regions on the left

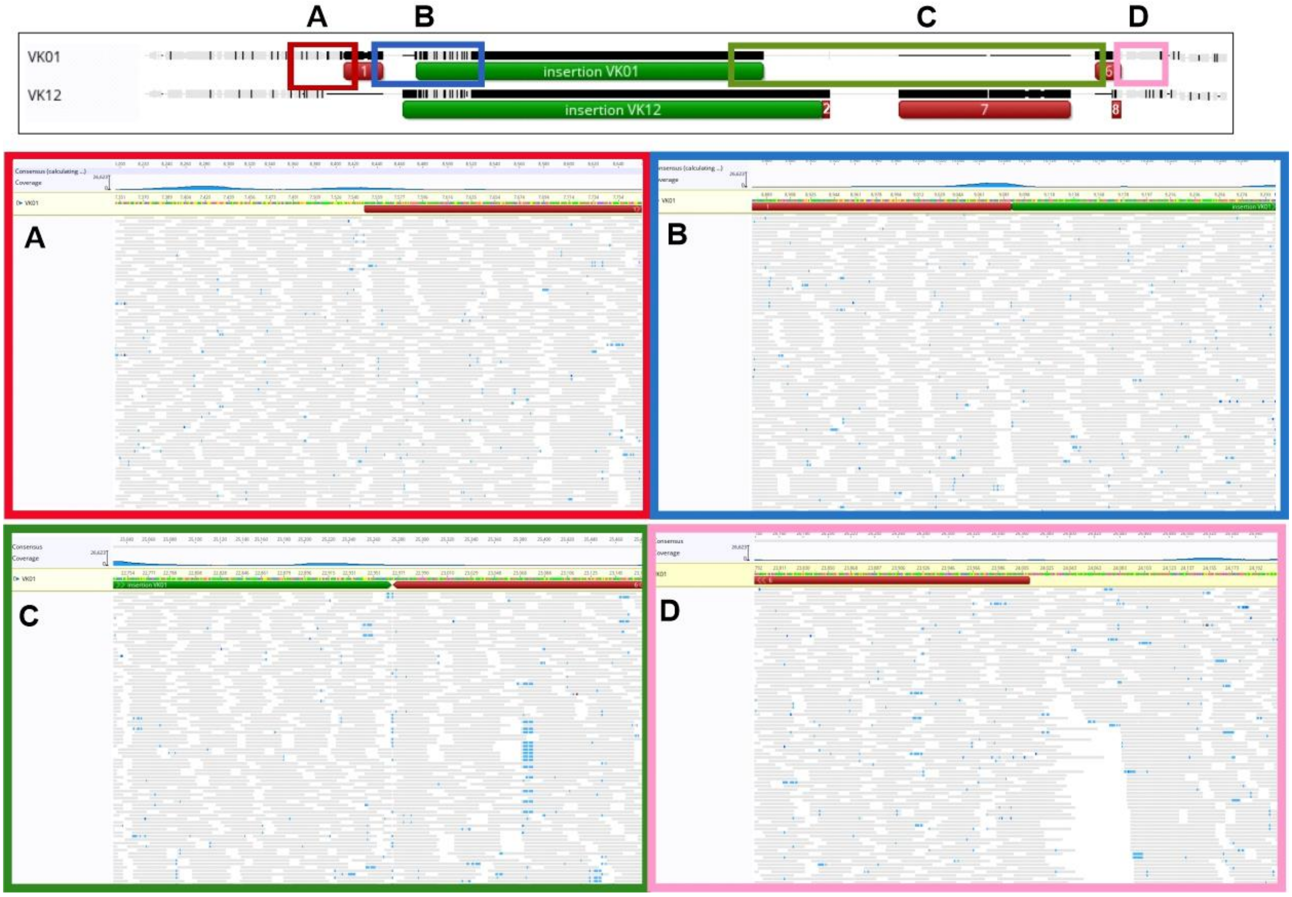

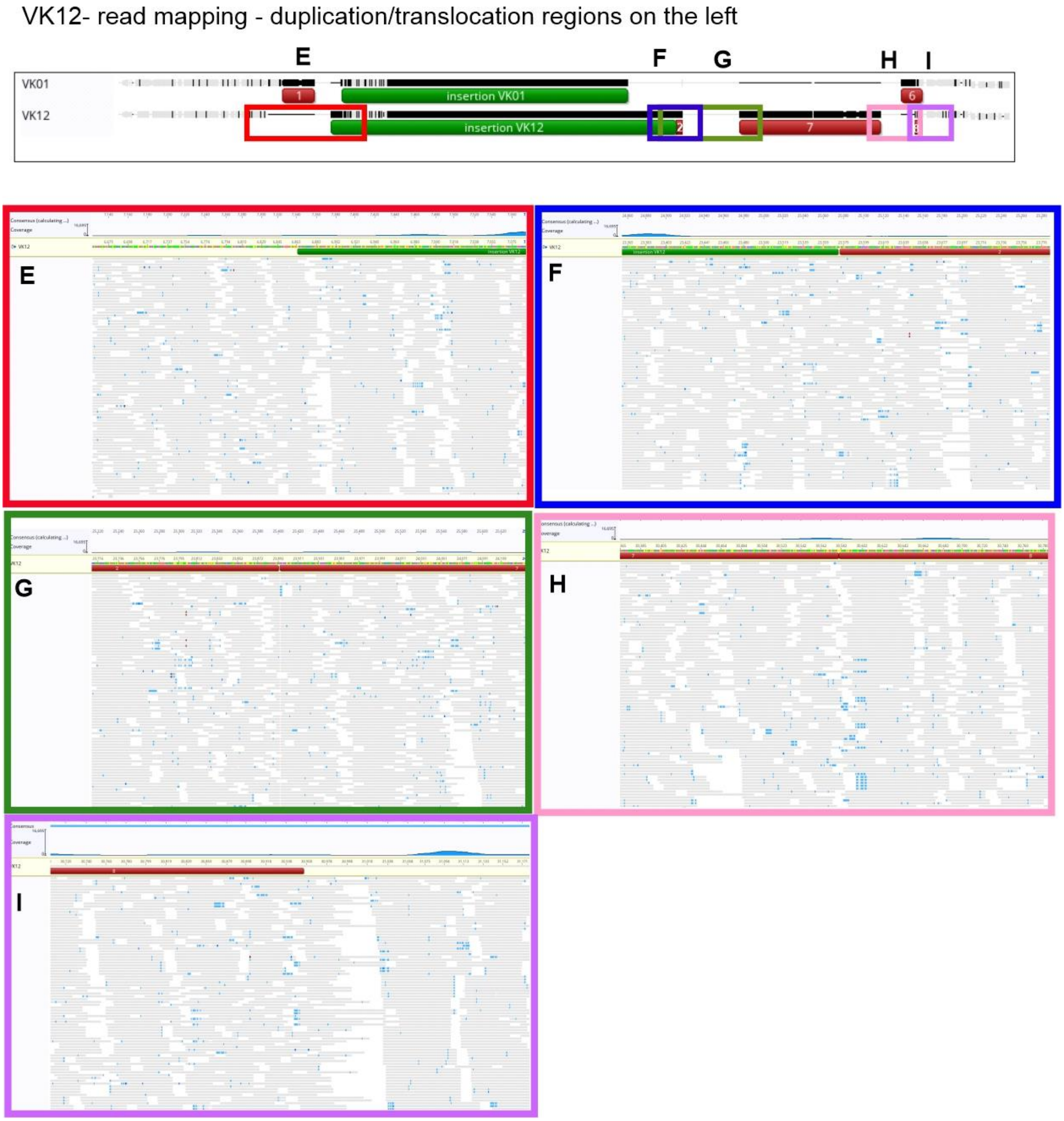

Figure S2. Mapping of VK01 (A-D) and VK12 (E-I) reads to the regions of long insertions in VK01 and VK12 genomes. (**A – I**) Top schemes show the long insertions of VK01 and VK12 (green bars) and fragments 1, 2, 6, 7, and 8 of the 10.7 kb insertion of HPXV (red bars) at the left end of the genomes, as shown in Figure 3A. Each region of transition is indicated by colored boxes and letters. Mapping of VK01 (**A-D**) and VK12 (**E to I**) reads to each transition region was performed using Geneious Prime and is shown below the schemes. A low coverage region was observed downstream region 6 in VK01 genome (**D**) and region 8 in VK12 genome (**I**). This low coverage region corresponds to AT-rich sequences located in the intergenic region between HPXV-15b and C11R genes (Figure S7G) and were detected in all VK genomes. HXPV-15b is the last gene of the 10.7 kb insertion at the left end of the HPXV genome (Figures 3B and 3C).

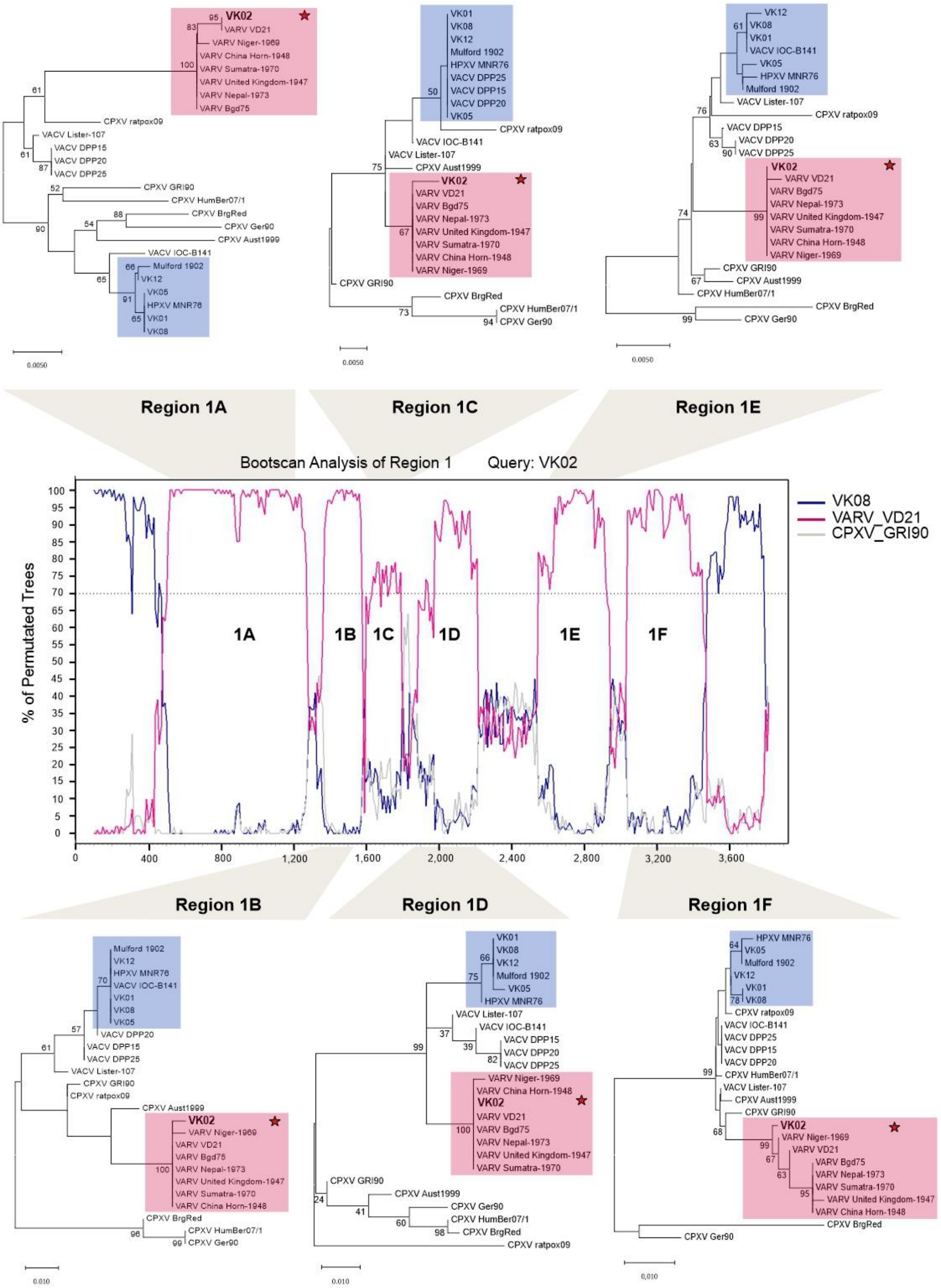

Figure S3. Bootscan analysis of region 1 to detect recombination subregions in the VK02 genome. Region 1 highlighted in Figure 7 was reanalyzed by Bootscan, using VK02 as query, window size 200, and step size 10. Six putative recombinant subregions with VARV were identified and marked from A to F. The VK02 sequences between recombination breakpoints were extracted and realigned with several orthopoxviruses for phylogenetic analysis, using either maximum-likelihood or neighbor-joining models. Blue boxes indicate the cluster containing HPXV and HPXV-related viruses and pink boxes indicate the VARV cluster. The position of VK02 in the trees is indicated by a red star. Numbers indicate the bootstrap support from 1,000 replicates (>50% is shown). The scale bar indicates the number of substitutions per site.

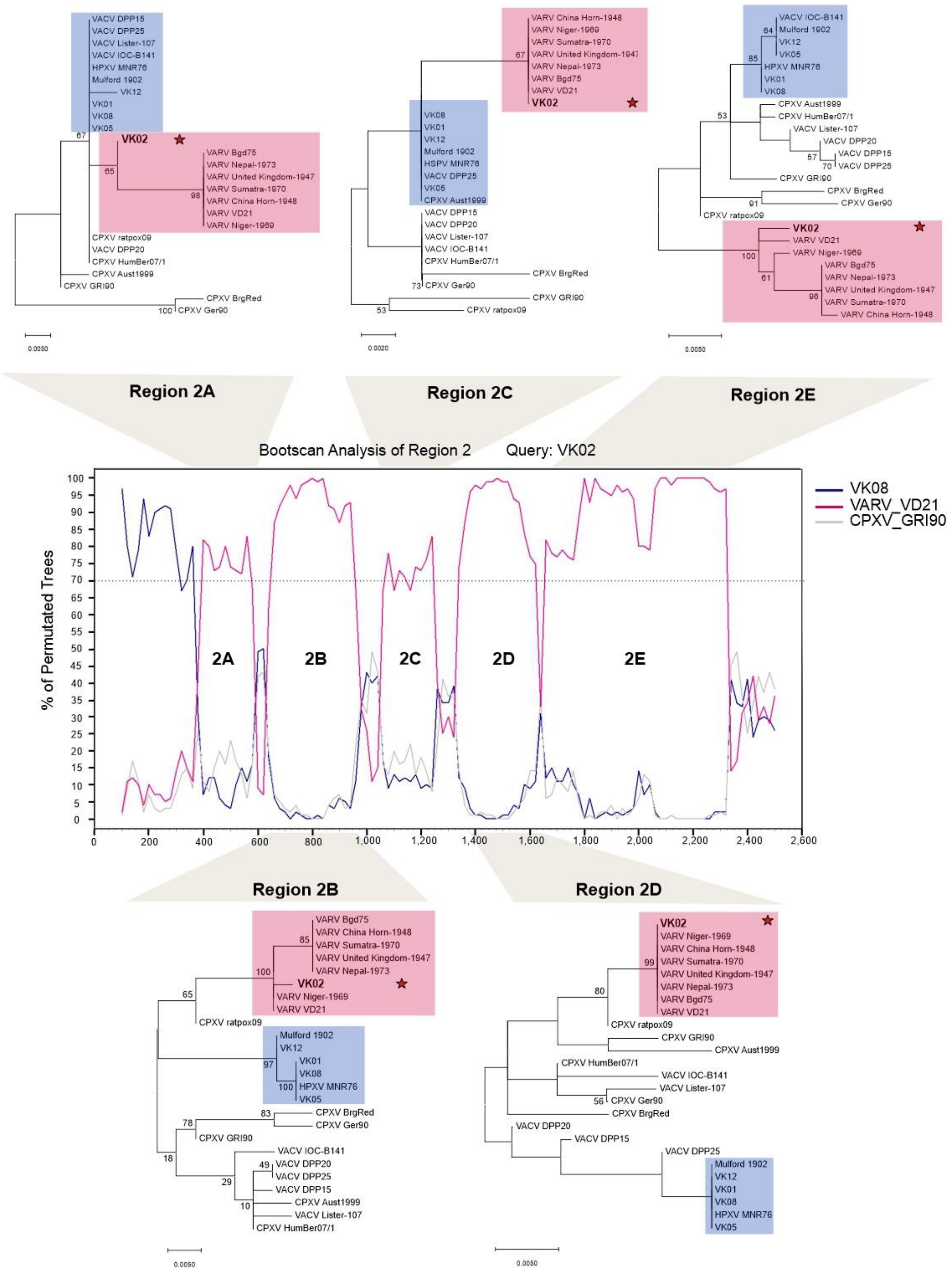

Figure S4. Bootscan analysis of region 2 to detect recombination subregions in the VK02 genome. Region 2 highlighted in Figure 7 was reanalyzed by Bootscan, using VK02 as query, window size 200, and step size 20. Five putative recombinant subregions with VARV were identified and marked from A to E. The VK02 sequences between recombination breakpoints were extracted and realigned with several orthopoxviruses for phylogenetic analysis using either maximum-likelihood or neighbor-joining models. Blue boxes indicate the cluster containing HPXV and HPXV-related viruses and pink boxes indicate the VARV cluster. The position of VK02 in the trees is indicated by a red star. Numbers indicate the bootstrap support from 1,000 replicates (>50% is shown). The scale bar indicates the number of substitutions per site.

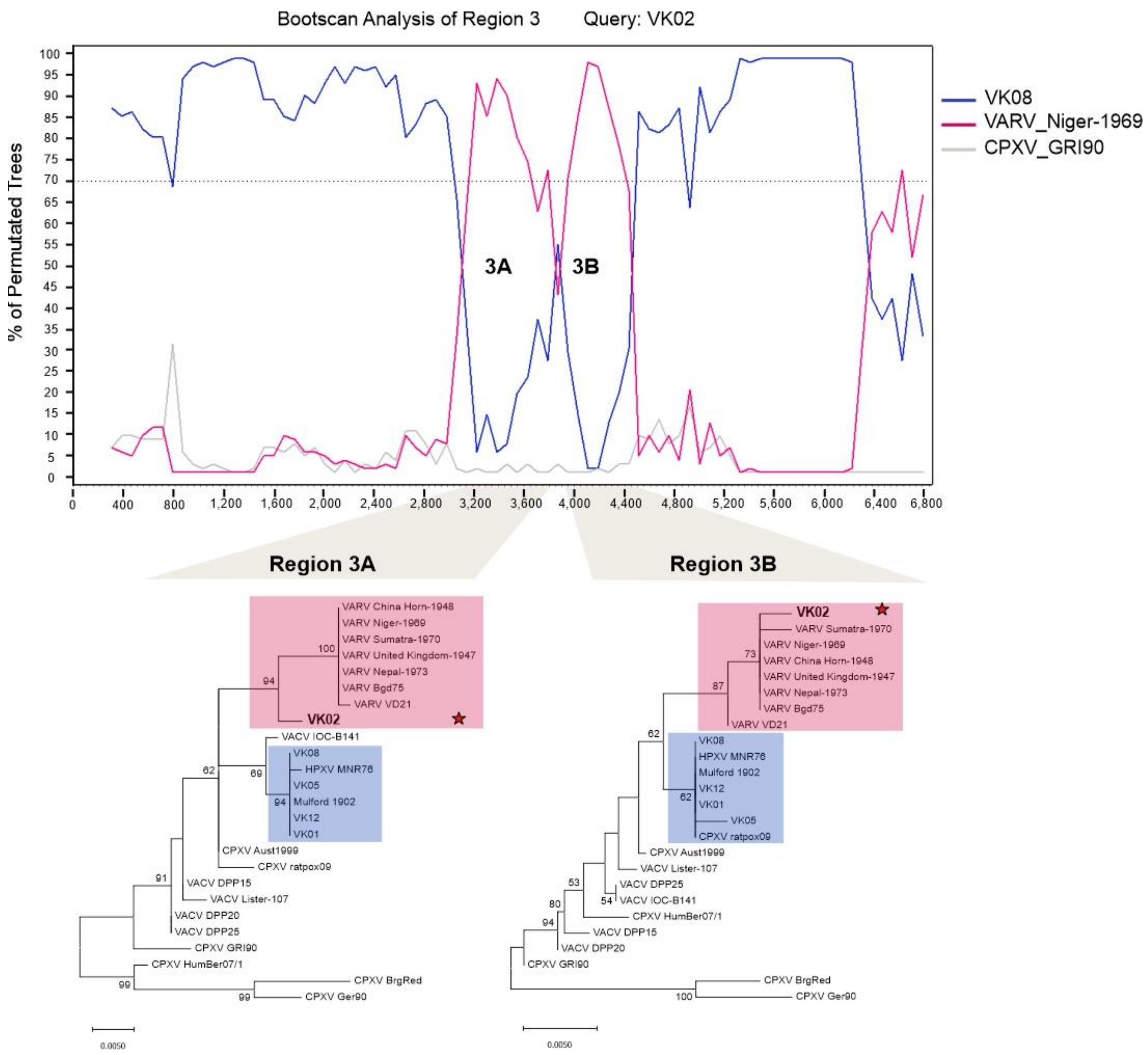

Figure S5. Bootscan analysis of region 3 to detect recombination subregions in the VK02 genome. Region 3 highlighted in Figure 7 was extracted and reanalyzed by Bootscan, using VK02 as query, window size 600, and step size 80. Two putative recombinant subregions with VARV were identified and marked A and B. The VK02 sequences between recombination breakpoints were extracted and realigned with several orthopoxviruses for phylogenetic analysis using either maximum-likelihood or neighbor-joining models. Blue boxes indicate the cluster containing HPXV and HPXV-related viruses and pink boxes indicate the VARV cluster. The position of VK02 in the trees is indicated by a red star. Numbers indicate the bootstrap support from 1,000 replicates (>50% is shown). The scale bar indicates the number of substitutions per site.

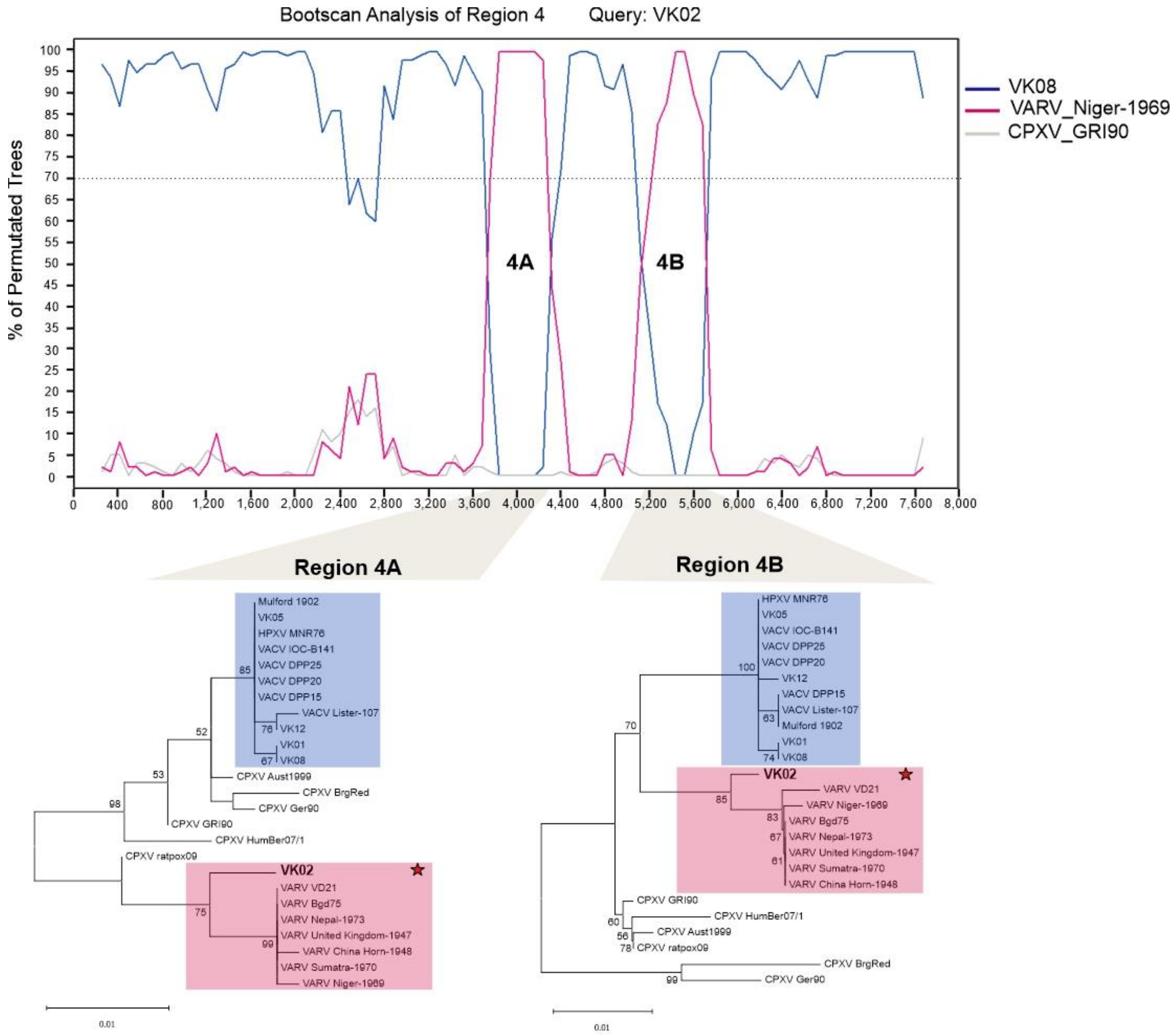

Figure S6. Bootscan analysis of region 4 to detect recombination subregions in the VK02 genome. Region 4 highlighted in Figure 7 was extracted and reanalyzed by Bootscan, using VK02 as query, window size 500, and step size 80. Two putative recombinant subregions with VARV were identified and marked A and B. The VK02 sequences between recombination breakpoints were extracted and realigned with several orthopoxviruses for phylogenetic analysis using either maximum-likelihood or neighbor-joining models. Blue boxes indicate the cluster containing HPXV and HPXV-related viruses and pink boxes indicate the VARV cluster. The position of VK02 in the trees is indicated by a red star. Numbers indicate the bootstrap support from 1,000 replicates (>50% is shown). The scale bar indicates the number of substitutions per site.

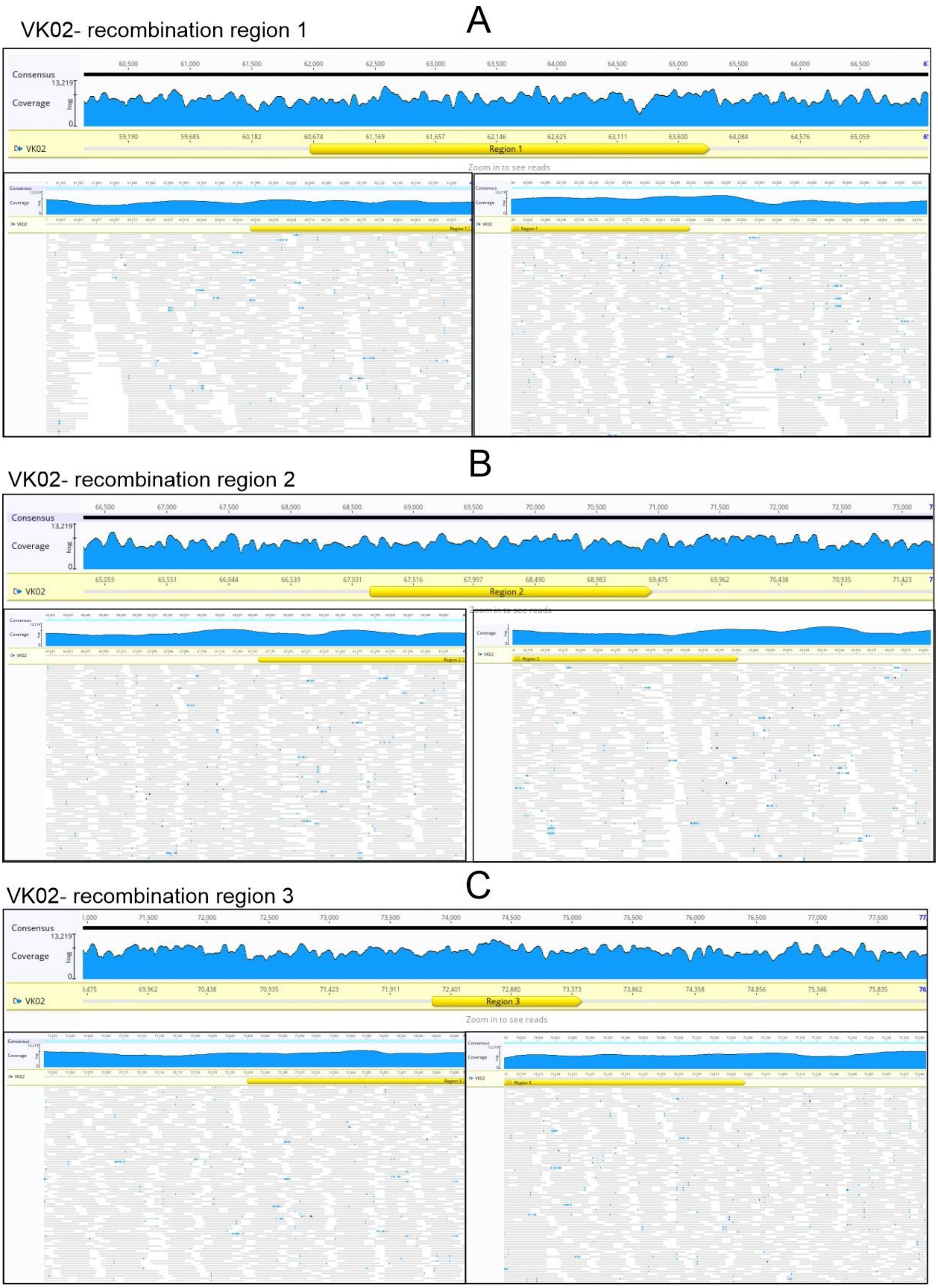
A
VK02- recombination region 1
Consensus
13,219
Coverage
log
0
60,500 61,000 61,500 62,000 62,500 63,000 63,500 64,000 64,500 65,000 65,500 66,000 66,500
59,190 59,685 60,182 60,674 61,169 61,657 62,146 62,625 63,111 63,600 64,084 64,576 65,059
VK02
Region 1
Zoom in to see reads
B
VK02- recombination region 2
Consensus
13,219
Coverage
log
0
66,500 67,000 67,500 68,000 68,500 69,000 69,500 70,000 70,500 71,000 71,500 72,000 72,500 73,000
65,059 65,551 66,044 66,539 67,031 67,516 67,997 68,490 68,983 69,475 69,962 70,438 70,935 71,423
VK02
Region 2
C
VK02- recombination region 3
Consensus
13,219
Coverage
log
0
71,500 72,000 72,500 73,000 73,500 74,000 74,500 75,000 75,500 76,000 76,500 77,000 77,500
69,962 70,438 70,935 71,423 71,911 72,401 72,880 73,373 73,862 74,358 74,856 75,346 75,835
VK02
Region 3
Zoom in to see reads

VK02- recombination region 4 D

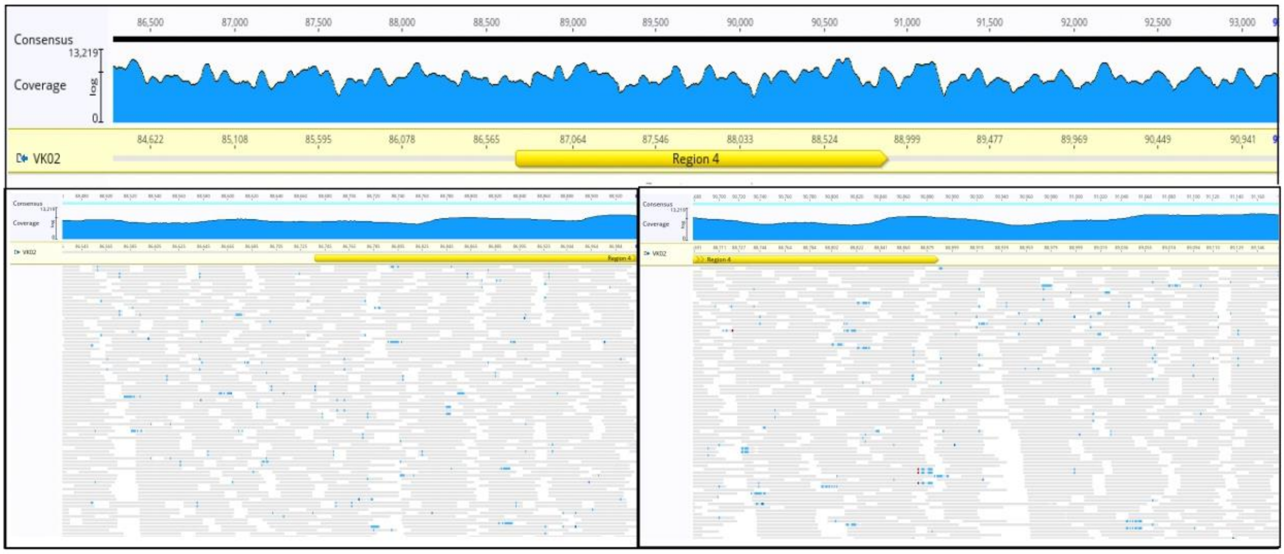

VK02- recombination region 5 E

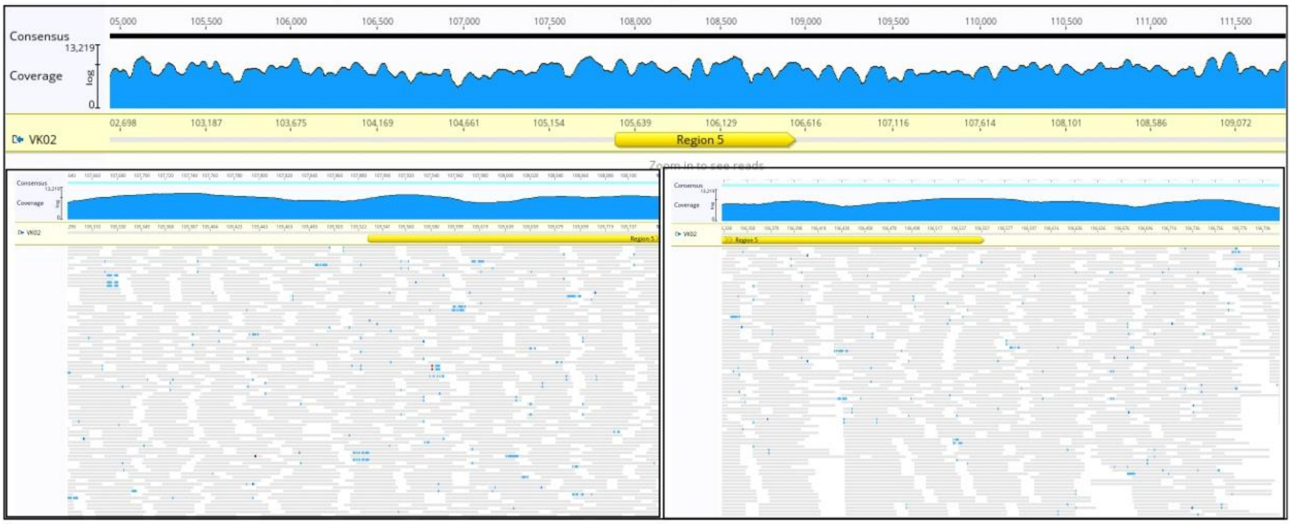

VK02- recombination region 6 F

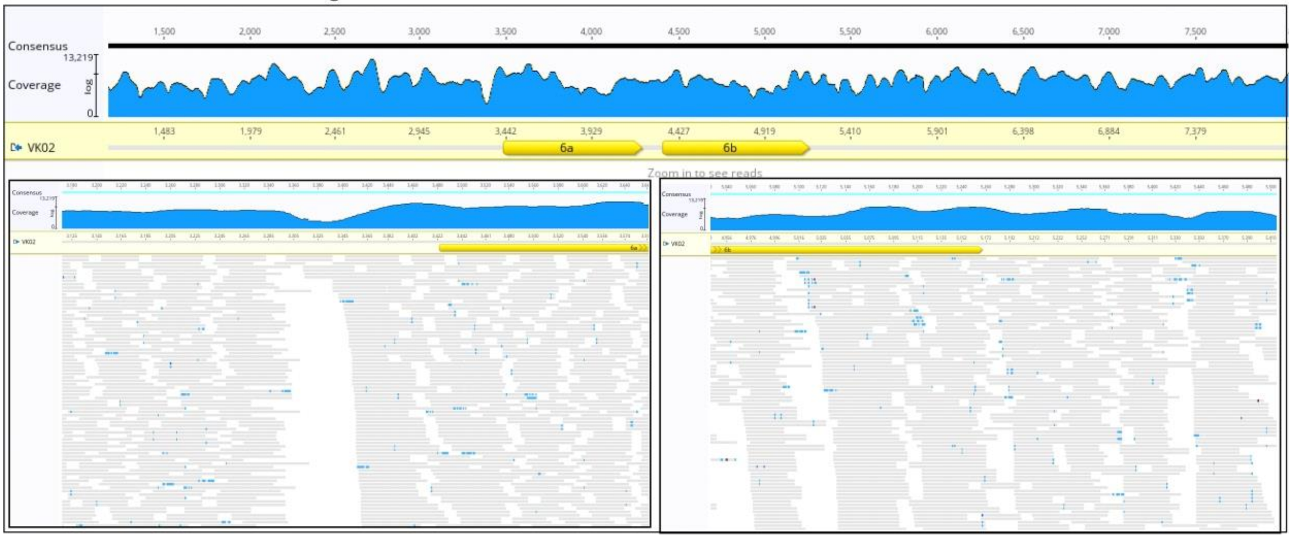

96

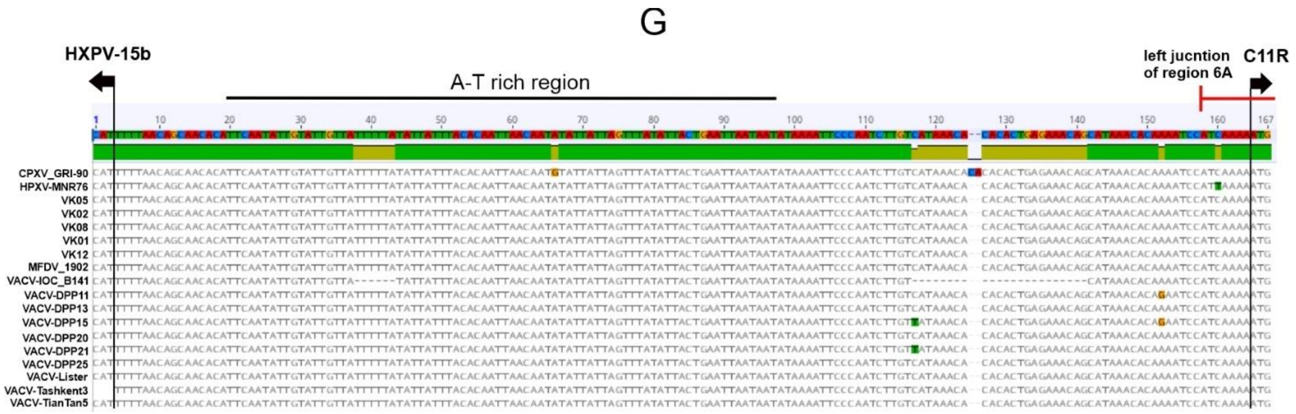

Figure S7. Mapping of VK02 (A-F) reads to the recombination regions of VK02 genome. (**A – F**) The six recombination regions of VK02 (yellow arrows) and coverage profile are shown on the top panel. Bottom panels show the left and right boundaries of each recombination region. (**G**) An alignment of the intergenic region between HPXV-15b and C11R genes is shown. The left arrow indicates the end of HPXV-15b gene, and the right arrow indicates the beginning of the C11R gene. The AT-rich region is indicated by a black bar on the top. The left boundary of recombination region 6A is indicated with a red bar.

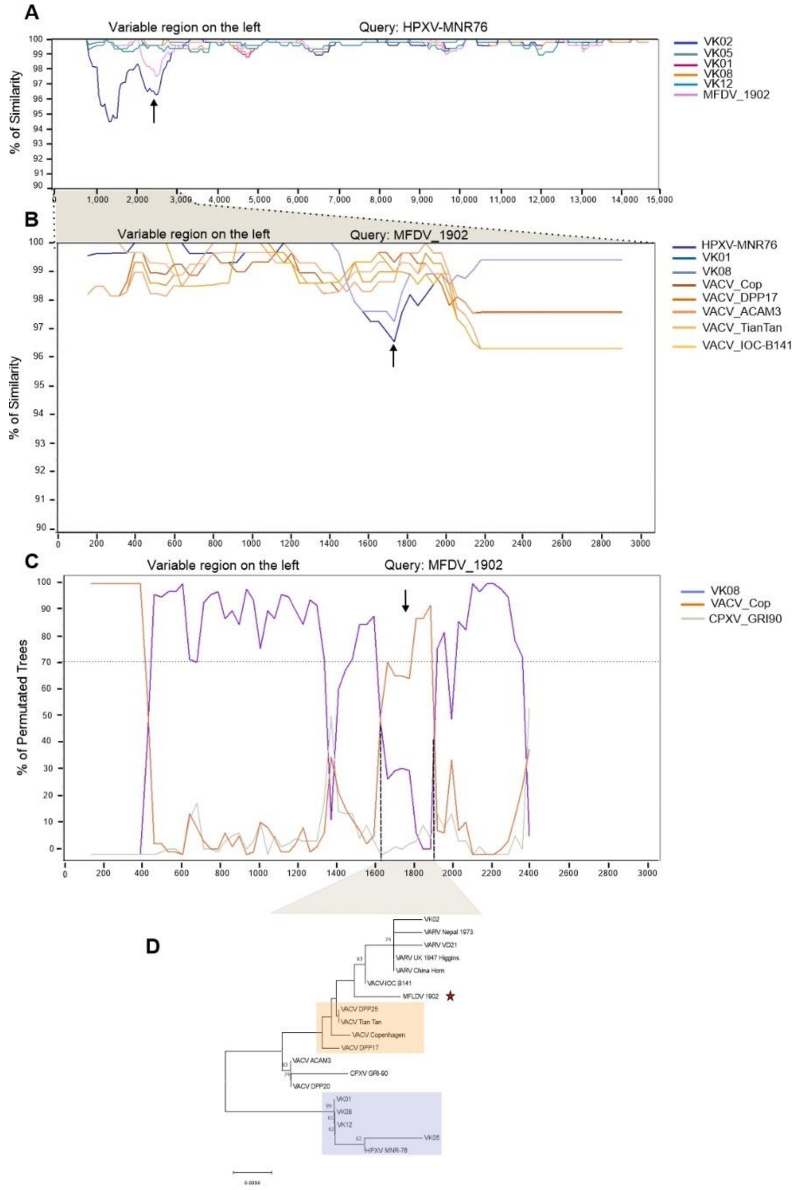

Figure S8. Similarity profile and Bootscan analysis to detect recombination events in the left variable region of the MFDV_1902 genome. A) The multialignment of the left variable region of the six historical smallpox vaccines and the HPXV_MNR-76 genomes was scanned for similarity analysis using Simplot, using HPXV_MNR-76 as query, window size 500, step size 50. A black arrow indicates the region of least similarity between MFDV_1902 and HPXV_MNR-76. **B)** The initial 3 kb segment of the multialignment was used in Simplot with window size 300, step size 40, and MFDV_1902 was selected as the query. The black arrow indicates the region of the least similarity between MFDV_1902 and both HPXV and VK08/VK01. **C)** The multialignment shown in **B**, was analyzed by Bootscan, using MFDV_1902 as query, window size 300, and step size 40. A putative recombinant region with VARV was identified (black arrow). The MFDV_1902 sequence between recombination breakpoints was realigned with several orthopoxviruses for phylogenetic analysis **(D)** using either maximum-likelihood or neighbor-joining models. Blue boxes indicate the HPXV cluster and orange boxes indicate the VACV cluster. The position of MFDV_1902 in the tree is indicated by a red star. Numbers indicate the bootstrap support from 1,000 replicates (>50% is shown). The scale bar indicates the number of substitutions per site.

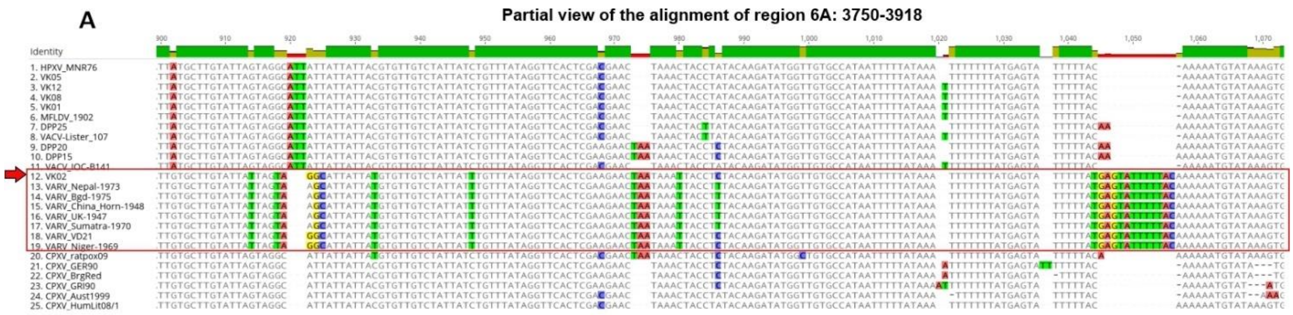

B

Bootscan regions and subregions	VACV-Cop Ortholog Region	Breakpoints in VK02 genome	Sequence size (bp)
#1	I5L, I6L, I7L, I8R	60648-63845	3,198
#1A	I5L, I6L	60747-61557	811
#1B	I6L	61596-61856	261
#1C	I6L	61866-62066	201
#1D	I7L	62146-62486	341
#1E	I7L	62495-63215	721
#1F	I8R	63296-63736	441
#2	G3L, G2R, G4L, G5R	67168-69418	2,251
#2A	G3L	67249-67427	179
#2B	G2R	67467-67807	341
#2C	G2R	67909-68068	160
#2D	G4L	68166-68466	301
#2E	G5R	68487-69167	681
#3	G8R, G9R, L1R	72217-73577	1,361
#3A	G8R, G9R	72217-72937	720
#3B	G9R, L1R	73017-73577	561
#4	H4L, H5R	86737-88888	2,152
#4A	H4L	86827-87386	560
#4B	H5R	88178-88889	712
#5	D13L, A1L	105530-106559	1,040
#6A	C11R, C10L	3422-4260	838
#6B	C10L	4300-5136	837

Figure S9. Details of the recombination events with VARV in the VK02 genomes. A) part of the nucleotide alignment of region 6A that contains the ortholog of C11R. Note the similar patterns of SNPs, insertions, and deletions in the VK02 and VARV genomes. These patterns are missing in other historical smallpox vaccines. **B)** location of the recombinant breakpoints, size of the recombinant regions and subregions, and gene *loci* where the recombination event occurred.

Table S1. List of genome sequences used in this work.

Virus	GenBank accession number
Historical vaccine Mulford_1902	MF477237
Historical vaccine VK01	BK013339
Historical vaccine VK02	BK013340
Historical vaccine VK05	BK013341
Historical vaccine VK08	BK013342
Historical vaccine VK012	BK013343
Horsepox (HPXV)_MNR-76	DQ792504
Vaccinia (VACV)_IOC-B141	KT184690
VACV_IOC-B388	KT184691
VACV_Serro 2	KF179385
VACV_Cantagalo isolate CM-01	KT013210
VACV_Cantagalo isolate CG-04	MW018155
VACV_Cantagalo isolate VI-04	MW018154
VACV_Cantagalo isolate MI-233	MW018153
VACV_Cantagalo isolate ALE-H2	MW018156
VACV_Wyeth clone A111	OP751801
VACV_Wyeth clone A211	OP751802
VACV_Wyeth clone A311	OP751803
VACV_Dryvax clone DPP11	JN654978
VACV_Dryvax clone DPP13	JN654980
VACV_Dryvax clone DPP15	JN654981
VACV_Dryvax clone DPP17	JN654983
VACV_Dryvax clone DPP20	JN654985
VACV_Dryvax clone DPP21	JN654986
VACV_Dryvax clone DPP25	KJ125438
VACV_Dryvax clone ACAM2000	AY313847
VACV_TianTan clone TP5	KC207811
VACV_TianTan clone TT9	JX489136
VACV_WR	AY243312
VACV_Lister	KF866253
VACV_Lister-LC16m8	AY678275
VACV_Lister-107	DQ121394
VACV_Tashkent clone TKT3	KM044309
VACV_Tashkent clone TKT4	KM044310
Buffalopox strain Karachi_2005	MG599038
Cowpox (CPXV)_GRI-90	X94355
CPXV_Finland_2000	HQ420893
CPXV_GER80 EP4	HQ420895
CPXV_GER2002 MKY	HQ420898
CPXV_Brighton-Red	AF482758
CPXV_France_Nancy-2001	HQ420894
CPXV_GER2010 MKY	LT896721
CPXV_GER-MygEK 938/17	LR812035
CPXV_HumAac09/1	KC813508
CPXV_HumGra07/1	KC813510
CPXV_Ratpox09	LN864565
Variola (VARV)_Bangladesh-1975	DQ437581
VARV_Brazil_Garcia-1966	DQ441419
VARV_Nepal-1973	DQ437588
VARV_China_Horn-1948	DQ437582
VARV_Sumatra-1970	DQ437591
VARV_UK-1947	DQ441446
VARV_South_Africa-1965	DQ441436
VARV_V563-1939-1969	LT706528
VARV_V1588-1809–1889	LT706529
VARV_Japan_Harper-1951	DQ441430
VARV_India-1953	DQ441428
VARV_Guinea-1969	DQ441426
VARV_Sierra_Leone-1969	DQ441437
VARV_Benin-1968	DQ441416
VARV_Niger-1969	DQ441434
VARV_VD21	BK010317

Table S2. Representative fragmentation pattern of genes located in different genomic regions of cowpox virus, horsepox virus, the historical smallpox vaccines, and some VACV strains.

HPXV Gene (VACV_Cop)		SIZE OF VIRUS GENES/ORFs (bp)												
	CPXV_BR (GRI-90 when different)	HPXV_ MNR-76	VK05	VK01	VK02	VK08	VK12	MFDV_ 1902	VACV_IOC -B141	VACV_ DPP25	VACV_ Lister	VACV_Tian Tan-03	VACV_ Tashkent-TK3	VACV_WR
**LEFT END REGION**														
**HPXV003 (CrmB)**	1068 (1056)	1050	1050	1050		1050	1050	411+384	192+105+360	285	192+255	360	369	369
**HPXV004**	1860 (1761)	1764	1854	1854		1854	1854	150+342+339+234+414	150+201+141+186+429+702	336+387+432+453	330+282	360+504	342+330+753	
**HPXV005a/203c**	2019	276	276	276		276	276	117						
**HPXV005b/203b (C18L/B24R)**		453	453	453		453	453	453	186	453	150	504		
**HPXV005c/203a (C17L/B23R)**		1194	1239	1239		1239	1236	1164	318+192+702	1149	1278			
**HPXV11a**	2295	792	795											
**HPXV11b**		867	867											
**HPXV11c**		450	450				450							
**HPXV14a**	2391 (2502)	282	282			309								
**HPXV14b**		747	1509		753	1509	882							
**HPXV14c**		612			819									
**HPXV14d**		498	498	549	498	498								
**HPXV15a**	513	222	222	222	222	222								
**HPXV15b**		249	249	282	282	282	282							

<table>
<tr><td>HPXV022 (C9L)</td><td>1899 (1848)</td><td>1905</td><td>1905</td><td>1905</td><td>1905</td><td>1905</td><td>1905</td><td>924+888</td><td>924+153+ 684</td><td>771+894</td><td>1905</td><td>1905</td><td>1905</td><td>1905</td></tr>
<tr><td>HPXV026a (C5L)</td><td rowspan="2">378 (612)</td><td>195</td><td rowspan="2">606</td><td rowspan="2">606</td><td rowspan="2">606</td><td rowspan="2">606</td><td rowspan="2">606</td><td rowspan="2">606</td><td rowspan="2">606</td><td rowspan="2">606</td><td rowspan="2">615</td><td rowspan="2">606</td><td rowspan="2">606</td><td rowspan="2">615</td></tr>
<tr><td>HPXV026b (C5L)</td><td>366</td></tr>
<tr><td>HPXV027a (C4L)</td><td>951</td><td>414</td><td rowspan="2">414+123+ 204</td><td rowspan="2">414+123+ 204</td><td rowspan="2">414+123+ 204</td><td rowspan="2">414+123+ 204</td><td rowspan="2">414+123+ 204</td><td rowspan="2">414+123+ 204</td><td rowspan="2">189+231+ 123+120</td><td rowspan="2">189+231+ 180</td><td rowspan="2">951</td><td rowspan="2">189+768</td><td rowspan="2">951</td><td rowspan="2">951</td></tr>
<tr><td>HPXV027b (C4L)</td><td></td><td>204</td></tr>
<tr><td>HPXV028 (C3L)</td><td>792</td><td>792</td><td>798</td><td>393+276</td><td>792</td><td>393+276</td><td>786</td><td>786</td><td>393+189</td><td>786</td><td>792</td><td>792</td><td>792</td><td>792</td></tr>
<tr><td>HPXV029 (C2L)</td><td>1539</td><td>1539</td><td>1539</td><td>1326+96</td><td>1539</td><td>1326+96</td><td>1539</td><td>1539</td><td>534+813</td><td>1521</td><td>1539</td><td>1539</td><td>1539</td><td>1539</td></tr>
<tr><td>HPXV033a (M1L)</td><td rowspan="3">1422</td><td>219</td><td>219</td><td>219</td><td>219</td><td>219</td><td>219</td><td>219</td><td>219</td><td rowspan="3">219+1197</td><td rowspan="3">1413</td><td rowspan="3">1413</td><td rowspan="3">1419</td><td rowspan="3">1419</td></tr>
<tr><td>HPXV033b (M1L)</td><td>654</td><td>654</td><td>654</td><td>654</td><td>654</td><td>654</td><td>654</td><td>654</td></tr>
<tr><td>HPXV033c (M1L)</td><td>480</td><td>480</td><td>504</td><td>504</td><td>405</td><td>405</td><td>405</td><td>474</td></tr>
<tr><td>HPXV034 (M2L)</td><td>663</td><td>663</td><td>663</td><td>663</td><td>492</td><td>663</td><td>663</td><td>663</td><td>663</td><td>663</td><td>663</td><td>663</td><td>663</td><td>663</td></tr>
<tr><td>HPXV035 (K1L)</td><td>855</td><td>855</td><td>855</td><td>855</td><td>855</td><td>855</td><td>855</td><td>855</td><td>855</td><td>855</td><td>855</td><td>855</td><td>855</td><td>855</td></tr>
<tr><td>HPXV036 (K2L)</td><td>1122</td><td>1122</td><td>1122</td><td>1122</td><td>1122</td><td>1122</td><td>1122</td><td>1122</td><td>1095</td><td>1095</td><td>1110</td><td>1110</td><td>1110</td><td>1110</td></tr>
<tr><td>HPXV037 (K3L)</td><td>267</td><td>267</td><td>267</td><td>267</td><td>267</td><td>267</td><td>267</td><td>267</td><td>183+183</td><td>267</td><td>267</td><td>267</td><td>267</td><td>267</td></tr>
<tr><td>HPXV038 (K4L)</td><td>1275</td><td>1275</td><td>1275</td><td>1275</td><td>1275</td><td>1275</td><td>1275</td><td>1275</td><td>1275</td><td>1275</td><td>1275</td><td>1275</td><td>1275</td><td>1275</td></tr>
</table>

**HPXV039 (K5L/K6L)**	831	690	834	135+630	135+630	135+630	135+630	135+366+312	516+234	516+195	135+405+246	366+255	345+246	405+246
**HPXV041 (F1L)**	756	765	765	723	723	723	756	690	462+207	681	681	681	681	681
**HPXV051 (F11L)**	1065	1065	1065	1065	1065	1065	1065	1065	1047	1047	1065	1065	1065	1047
**HPXV062 (E5R)**	960 (996)	996	996	1026	996	996	996	996	996	507+450	996	996	996	1026
**HPXV074a (I4L)**	2316	243	1245+858+291	1245+861+291	1245+861+291	1245+861+291	1245+861+291	1221+861+291	2316	2316*	2316	2316	2316	2316
**HPXV074b (I4L)**		1323												
**HPXV074c (I4L)**		375												
**HPXV074d (I4L)**		291												
**HPXV091 (L2R)**	267 (279)	264	264	264	264	264	264	258	258	258	264	264	258	264
**RIGHT END REGION**														
**HPXV146a (A25L)**	3855 (3840)	213	213	792+165	213+477+165	612+165	213+612+165	213	180	198+465	639	702	243+465	198+465
**HPXV146b (A25L)**		612	612					465						
**HPXV146c (A25L)**		699	699	699	699	699	699	684	684	684	684	684	681	684
**HPXV146d (A25L)**		2178	2178	2178	2178	2178	2178	2178	2169	2166	2178	2178	2178	2178

<table>
<tr><td>HPXV161 (A39R)</td><td>1230 (1209)</td><td>981</td><td>981</td><td>990+123</td><td>891+84</td><td>411+123</td><td>159+108+ 106</td><td>684+207+ 123</td><td>681+258+ 123</td><td>795+429</td><td>1212</td><td>774</td><td>734+429</td><td>888</td></tr>
<tr><td>HPXV173a (A51R)</td><td rowspan="2">1005</td><td>243</td><td>243</td><td>252</td><td>258</td><td>252</td><td rowspan="2">921</td><td rowspan="2">1005</td><td>210</td><td>210</td><td rowspan="2">1005</td><td rowspan="2">1005</td><td rowspan="2">1017</td><td rowspan="2">1005</td></tr>
<tr><td>HPXV173b (A51R)</td><td>813</td><td>813</td><td>801</td><td>807</td><td>801</td><td>540</td><td>801</td></tr>
<tr><td>HPXV178a (A57R)</td><td rowspan="2">594</td><td>189</td><td rowspan="2">594</td><td rowspan="2">594</td><td rowspan="2">594</td><td>114</td><td>114</td><td>114</td><td>171</td><td>114</td><td></td><td></td><td>114</td><td></td></tr>
<tr><td>HPXV178b (A57R)</td><td>294</td><td>294</td><td>294</td><td>456</td><td>456</td><td>456</td><td>456</td><td>456</td><td>456</td><td>456</td></tr>
<tr><td>HPXV183a (B6R)</td><td rowspan="2">540 (552)</td><td>336</td><td rowspan="2">534</td><td rowspan="2">534</td><td rowspan="2">534</td><td rowspan="2">534</td><td rowspan="2">534</td><td rowspan="2">522</td><td rowspan="2">522</td><td rowspan="2">522</td><td rowspan="2">522</td><td rowspan="2">522</td><td rowspan="2">522</td><td rowspan="2">522</td></tr>
<tr><td>HPXV183b (B6R)</td><td>198</td></tr>
</table>

* In DPP17, the ortholog of I4L is split between two ORFs of 1251 bp and 1152 bp.

Table S3. Blastn identity scores between putative recombinant regions in VK02 genome and VARV strains[a].

VARV strain	VK02 genome					
	**Region #1**	**Region #2**	**Region #3**	**Region #4**	**Region #5**	**Region #6**
VD21_17th century	**99.72%**	**99.19%**	99%	98.24%	**99.71%**	98.45%
Sumatra 1970	99.59%	98.92%	**99.17%**	98.19%	99.62%	99.25%
V563_1939-1969	99.59%	98.92%	**99.17%**	98.19%	99.62%	99.25%
Nepal 1973	99.59%	98.89%	**99.17%**	98.14%	99.62%	99.14%
China Horn 1948	99.59%	98.89%	**99.17%**	98.1%	99.62%	99.19%
Bangladesh 1975	99.59%	98.89%	**99.17%**	98.14%	99.62%	99.19%
United Kingdom 1947	99.59%	99.15%	**99.17%**	98.19%	99.62%	99.25%
South Africa 1965	99.59%	98.92%	**99.17%**	98.14%	99.62%	99.25%
Japan-Harper 1951	99.59%	98.92%	**99.17%**	97.92%	99.62%	98.56%
India 1953	99.59%	98.92%	**99.17%**	98.14%	99.62%	99.25%
V1588_ 1809–1889	99.53%	99.15%	99.08%	98.19%	99.52%	99.25%
Guinea 1969	99.53%	99.15%	99.08%	98.14%	99.52%	99.31%
Benin 1968	99.53%	99.15%	99.08%	98.14%	99.52%	**99.37%**
Sierra Leone 1969	99.5%	99.15%	99.08%	98.1%	99.52%	99.31%
Niger 1969	99.5%	99.15%	**99.17%**	**98.28%**	99.52%	**99.37%**
Brazil_Garcia 1966	99.47%	99.15%	99%	98.14%	99.52%	99.25%
range of % identity among the first 50 VARV hits	99.72%-99.09%	99.19%- 98.85%	99.17%-99%	98.28%-97.86%	99.71%-98.75%	99.37%-97.35%

[a]The highest identity scores are in bold.
